# Efficacy of different dosages of common uric acid-lowering medications in gout patients: a network meta-analysis of randomized control trials

**DOI:** 10.3389/fphar.2025.1565530

**Published:** 2025-06-25

**Authors:** Xiaojia Zheng, Cunxiang Xie, Jinying Fang, Zhenghui Huang, Jian Huang, Luming Zhao, Guancheng Ye, Hailong Wang

**Affiliations:** Department of Rheumatology, Dongzhimen Hospital, Beijing University of Chinese Medicine, Beijing, China

**Keywords:** gout, common uric acid-lowering medications, dosage, network meta-analysis, dose-response, RCTs

## Abstract

**Purpose:**

Urate lowering therapy (ULT) is extensively utilized for managing patients with gout. This study aims to compare the efficacy of different ULTs on serum uric acid (SUC) levels, gout flares, and adverse events (AEs) in gout patients.

**Methods:**

Studies comparing the efficacy of febuxostat, allopurinol, benzbromarone, and topixostat with placebo were searched up to March 2024. Stata 15.1 and R software 4.2.3 were employed to rank the efficacy of each ULT.

**Results:**

This study included 30 studies, involving 20,040 patients. All ULTs resulted in notably lower SUC levels compared to placebo/no ULT. Febuxostat 120 mg markedly reduced SUC levels compared to allopurinol and benzbromarone 25 mg (mean difference = 2.16, 95% confidence interval [0.27, 4.06], P < 0.05). Allopurinol 200/300 mg was the best choice to reduce gout flares. In terms of AEs, the allopurinol group (300 mg) had the lowest incidence of cardiovascular and renal abnormalities. Moreover, the incidence of AEs was observed to rise with increasing doses. Future well-designed randomized control trials are required to further confirm these findings.

**Conclusion:**

The study results indicate that febuxostat is the most effective ULT drug to treat gout. It can effectively help gout patients reduce SUC levels. Researchers should pay attention to the safety of drug doses.

## 1 Introduction

The most recent global burden of disease (GBD) estimates reveal that gout impacts 41 million individuals around the world (Dalbeth et al., 2021; He et al., 2023). According to a recent study, the global prevalence of gout ranges from 1% to 6.8%, while the incidence is between 0.58 and 2.89 per 1,000 people (Dehlin et al., 2020). As people’s material life and living standards continually improve, the increased intake of purines has led to a yearly rise in the prevalence of gout (Danve et al., 2021). This condition arises from persistently high serum uric acid (SUA) levels (hyperuricemia). It is marked by the deposition of monosodium urate (MSU) crystals in both articular and non-articular structures, like bursae, tendons, and entheses. Gout is the most prevalent cause of inflammatory arthritis (Chen et al., 2023). Currently, it has emerged as a major public health issue, inflicting noticeable harm, pain, and a substantial economic burden on patients (Chen et al., 2023; Danve and Neogi, 2020). Therefore, in clinical practice, there is an urgent need to treat gout and prevent its recurrence.

Long-term high SUA levels have long been regarded as the main pathological factor of gout (Shi et al., 2023). Hence, the American College of Rheumatology (ACR) guidelines for gout management (2020 edition) and the European League Against Rheumatism guideline for gout management (2016 edition) suggest the use of urate lowering therapy (ULT) as the first-line treatment for managing gout. Among ULTs, febuxostat, allopurinol, benzbromarone, and topiroxostat are the most often employed agents for lowering SUA levels (FitzGerald et al., 2020; Richette et al., 2016; Terkeltaub, 2023; Neilson et al., 2022; Anders et al., 2021; Zeng et al., 2023). Several previous meta-analyses (Wu et al., 2024; Gao et al., 2021; Salanti et al., 2011; Fan et al., 2021; Fan et al., 2020; Xie et al., 2023) have shown that both febuxostat and allopurinol are effective in reducing SUA levels in gout patients, Moreover, febuxostat exhibits greater efficacy than allopurinol. Fan et al. (2021) have found that febuxostat (80/120/240 mg/d) is more effective than allopurinol (200/300 mg once daily) in reducing SUA levels for gout patients, with favorable tolerance. As the dose of febuxostat increases, more patients achieve the target SUA level of less than 6.0 mg/dL. Fan et al. (2020) have found that titrating to a daily dose of 120 mg of febuxostat is superior to other doses in managing gout. Xie et al. (2023) have found that febuxostat 80 mg is effective in reducing high SUA levels to 6.0 mg/dL or less. However, this dosage does not show a better curative effect in reducing the incidence of gout compared to other treatments. Castrejon et al. (2015) have found that allopurinol is superior to benzbromarone in reducing SUA levels. Moreover, Lee and Song (2022a) have found that febuxostat 40 mg is more effective than benzbromarone 50 mg. Additionally, allopurinol is considered a safer alternative compared to other ULTs. However, further studies are needed to evaluate the safety of higher doses and long-term use. Lee and Song (2022b) have conducted a network meta-analysis (NMA) to compare the efficacy and safety of different doses of topiroxostat for treating gout with or without hyperuricemia. They find that topiroxostat 200 mg is the most effective option. Nevertheless, topiroxostat 200 mg would increase the risk of AEs. To the best of our knowledge, only two (Lee and Song, 2022a; Li et al., 2023) studies have compared the efficacy of different doses of common drugs for treating gout, with inconsistent results. Therefore, further exploration is still needed in the selection of ULTs, especially in dosage choices.

Although ULT is a proven approach for managing gout, there remains controversy over the choice of drugs and dosages, particularly regarding their effectiveness in treating gout flares. Despite previous studies confirming the efficacy and safety of ULTs, the relative effectiveness and safety of different doses remain unclear. This uncertainty limits the comprehensive ranking of these drugs for treating gout and other high uric acid-related conditions. Our purpose is to use an NMA to compare different gout medications and dosages, providing evidence-based data to guide clinical practice and improve the management of the condition.

## 2 Material and methods

### 2.1 Design and registration

Our NMA was performed in adherence to the Preferred Reporting Items for Systematic Reviews and Meta-analysis (PRISMA) statement. This study has been registered in the International Prospective Register of Systematic Reviews (identifier CRD42024549697).

### 2.2 Data source and search strategy

A systematic search was conducted in Web of Science, PubMed, EMBASE, and Cochrane Central Register of Controlled Trials databases up to 31 March 2024. Both MeSH terms and free-text terms were leveraged to search, including “gout”, “allopurinol”, “febuxostat”, “benzbromarone”, and “topiroxostat”. The search strategies are available in Supplementary Table S1. To ensure thorough coverage, reference lists of all relevant articles and reviews were also manually screened. The search was conducted by two investigators (Z.X.J and X.C.X). Moreover, another two investigators (F.J.Y. and H.Z.H.) reviewed uncertain studies. The ultimate decisions were made following a discussion.

### 2.3 Inclusion and exclusion criteria

The following studies were included: (i) participants (≥18 years) were diagnosed with gout according to 2015 ACR classification criteria and SUA levels ≥8.0 mg/dL; (ii) patients in the intervention group were treated with one or more drugs like febuxostat, allopurinol, benzbromarone, and topiroxostat, or combinations of above-mentioned drugs in any dosages; (iii) patients in the control group received a placebo or a different uricosuric agent compared to those in the intervention group; (iv) randomized controlled trials (RCTs) or clinical trials; (v) studies that reported at least one outcomes, including SUA levels, gout flare, and adverse events (AEs); (vi) studies that published in English.

The following studies were excluded: (i) studies that presented incomplete or erroneous data, and with no accessible original data; (ii) duplicate publications; (iii) patients with concomitant diseases, including cardiovascular disease and chronic renal disease; (iv) previously published meta-analysis, reviews, conference abstracts, animal experiments, case reports, and other non-clinical studies; (v) studies with unclear diagnostic and efficacy criteria.

### 2.4 Data extraction

Data extraction was independently performed by two investigators (Z.X.J. and X.C.X.) based on an extraction form. The extracted data included first author, publication year, basic characteristics of patients (baseline SUA levels), case numbers in each group, diagnosis criteria, treatment protocol, treatment duration (weeks), and study design. In this study, the outcome measures were SUA levels ≤6.0 mg/dL, the incidence of gouty flares, and the incidence of any AEs (such as abnormal liver function, renal impairment, cardiovascular disease, rash, and gastrointestinal disorders) during the period of the trial. These data were extracted by one investigator (Z.X.J.) and then confirmed by another investigator (X.C.X.).

### 2.5 Risk of bias (ROB) assessment

ROB in this study was assessed by two investigators (Z.X.J. and X.C.X.) through the Cochrane risk-of-bias tool in randomized trials (RoB 2) (Cumpston et al., 2019). Seven domains of bias were evaluated: selection bias related to randomization sequence generation, performance bias related to allocation concealment, performance bias related to blinding of participants and personnel, detection bias related to blinding of outcome assessment, withdrawal bias related to incomplete data, reporting bias related to selective reporting, and other bias. ROB in each domain was classified as high, low, or unclear risk. Studies with no explicit evidence of selection bias or other bias were rated as low or unclear risk. Disagreements were addressed through discussion with a third investigator (F.J.Y.).

### 2.6 Evidence quality assessment

The evidence quality was assessed with Grading of Recommendations Assessment, Development and Evaluation (GRADE). For each outcome measure, the quality of each evidence was rated as either high, moderate, low, or very low based on the ROB, inconsistency, indirectness, publication bias, intransitivity, incoherence (difference between direct and indirect effects), and imprecision. We made judgments of imprecision using the minimally contextualized approach and sourced minimally important differences for outcome measures either from the articles or through consensus by authors.

### 2.7 Data analysis

Stata 15.1 and R software 4.2.3 were employed to perform statistical analysis. According to the characteristics of variable type, the effect size that most accurately represented the overall data was chosen.

JAGS software (gemtc 0.8-2 and rjags 4–10 package) in R (version 4.1.2) (Rstudio, Boston, MA, United States) was leveraged to develop statistical models based on the Bayesian framework. The effect size for continuous variables was assessed using the mean difference (MD) and its 95% confidence interval (CI). A pooled risk ratio (RR) along with a 95% CI was computed for categorical variables. A random-effect model was employed for the NMA due to the clinical heterogeneity among the included studies (various countries, doses of ULT drug, treatment durations). For each outcome, four Markov chains were executed, with each chain yielding 50,000 iterations. Out of these, 20,000 iterations were discarded as the burn-in period. To assess convergence, both plots and the Gelman-Rubin-Brooks statistic were utilized (Brooks and Gelman, 1998). To estimate the relative rank of different ULTs for each outcome measure, the surface under the cumulative rank curve (SUCRA) was employed (Veroniki et al., 2016). A greater SUCRA value reflected a higher position in the intervention ranking (Veroniki et al., 2016). The deviance information criteria (DIC) was leveraged to compare the consistency and inconsistency models. A difference of less than 5 points in DIC indicated high consistency, leading to the construction of a consistency model (Dempster, 1997). To evaluate heterogeneity, the I^2^ statistic was employed. Heterogeneity was categorized as low for I^2^ values below 25%, moderate for values from 25% to 75%, and high for values exceeding 75% (Cumpston et al., 2019). Publication bias was examined through the use of comparison-adjusted funnel plots. Stata (version 17.0) (StataCorp, College Station, Texas, United States) was leveraged to create network plots and comparison-adjusted funnel plots for NMA.

In order to determine the overall and specific drug doses that lead to the most notable effect, also known as the optimal drug dose, we summarized the results of the dose-response relationship using model-based NMA (MBNMA). The MBNMAdose package (Pedder, 2021) was employed to perform MBNMA and dose-response analysis. The metacart package (Dusseldorp et al., 2014) was used to perform meta-analysis. The ggplot2 package (Wickham, 2011) was employed to plot dose-response curves. The code required to replicate the findings in this manuscript is available on the GitHub account of the primary author (URL: https://github.com/dgalgom/Physical-Activity-and-Cognitive-Function-Dose-response-Model-Based-Network-Meta-Analysis/blob/main/.github/workflows/blank.yml).

## 3 Results

### 3.1 Search results

**FIGURE 1:**
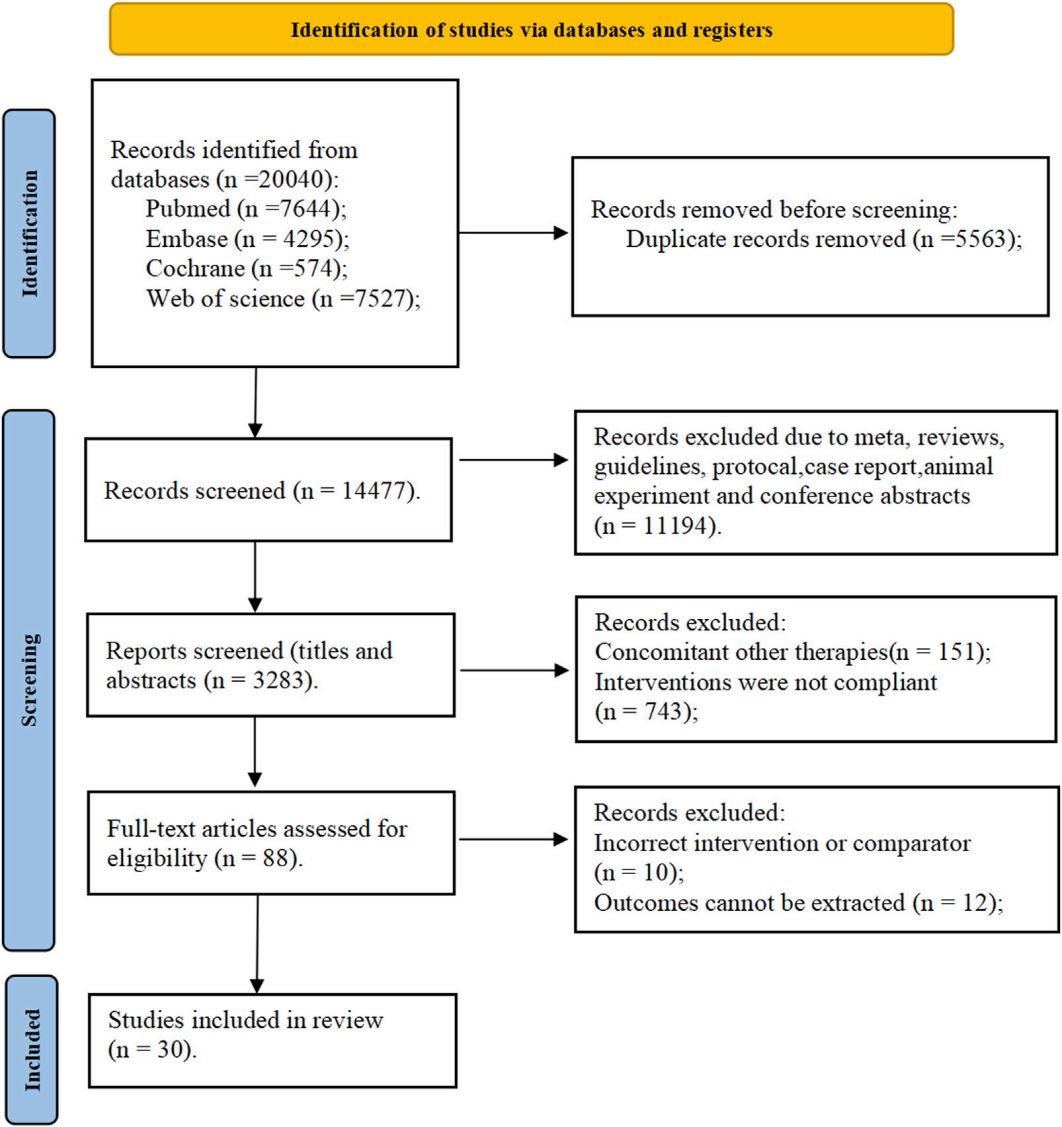
PRISMA flow diagram for search and selection of eligible studies included in the network meta-analysis.

The process of selecting studies is depicted in the PRISMA flowchart (Figure 1). 20,040 possibly relevant studies were initially identified from the four databases. After removing 5,563 duplicates, the title and abstract of each study were reviewed based on the established inclusion and exclusion criteria. At last, 14,389 studies were excluded, leaving 88 studies for full-text examination. Among the 88 articles, 58 studies were excluded due to failure to meet the ACR criteria for gout, or absence of outcome data. Finally, 30 studies were included in the NMA (Figure 1).

### 3.2 Characteristics of the included studies

The characteristics of each included study are presented in Table 1. Overall, 30 studies (Becker et al., 2009; Xu et al., 2015; Becker et al., 2010; Schumacher et al., 2008; Kamatani et al., 2011a; Wells et al., 2012; Kamatani et al., 2011b; Liang et al., 2019; Zhang et al., 2019; Lertnawapan and Jatuworapruk, 2021; Liu et al., 2022; O'Dell et al., 2022; Yan et al., 2022; Xue et al., 2024; Hosoya et al., 2016; Becker et al., 2005a; Kamatani et al., 2011c; Lee et al., 2023; Yamanaka et al., 2018; Schumacher et al., 2009; Kim et al., 2014; Hosoya et al., 2017; Reinders et al., 2009; Becker et al., 2005b; Saag et al., 2022; Mu et al., 2019; Jackson et al., 2012; Kojima et al., 2019; Saag et al., 2016; Peng et al., 2023) were included, involving 20,422 patients with gout. The included studies were sourced from almost every region worldwide, with 18 from China, Japan, Thailand, and Korea in Asia (Xu et al., 2015; Kamatani et al., 2011a; Kamatani et al., 2011b; Liang et al., 2019; Zhang et al., 2019; Lertnawapan and Jatuworapruk, 2021; Liu et al., 2022; Yan et al., 2022; Xue et al., 2024; Hosoya et al., 2016; Kamatani et al., 2011c; Lee et al., 2023; Yamanaka et al., 2018; Kim et al., 2014; Hosoya et al., 2017; Mu et al., 2019; Kojima et al., 2019; Peng et al., 2023), 11 from the United States in North America (Becker et al., 2009; Becker et al., 2010; Schumacher et al., 2008; Wells et al., 2012; O'Dell et al., 2022; Becker et al., 2005a; Schumacher et al., 2009; Hosoya et al., 2017; Saag et al., 2022; Mu et al., 2019; Saag et al., 2016), and one from the Netherlands in Europe (Reinders et al., 2009). Treatment durations for gout varied from 4 to 52 weeks. Most patients received treatments for 24–28 weeks. Febuxostat was reported in 28 studies (Becker et al., 2009; Xu et al., 2015; Becker et al., 2010; Schumacher et al., 2008; Kamatani et al., 2011a; Wells et al., 2012; Kamatani et al., 2011b; Liang et al., 2019; Zhang et al., 2019; Lertnawapan and Jatuworapruk, 2021; Liu et al., 2022; O'Dell et al., 2022; Yan et al., 2022; Xue et al., 2024; Becker et al., 2005a; Kamatani et al., 2011c; Lee et al., 2023; Yamanaka et al., 2018; Schumacher et al., 2009; Kim et al., 2014; Reinders et al., 2009; Becker et al., 2005b; Saag et al., 2022; Mu et al., 2019; Jackson et al., 2012; Kojima et al., 2019; Saag et al., 2016; Peng et al., 2023), allopurinol in 21 studies (Becker et al., 2009; Xu et al., 2015; Becker et al., 2010; Schumacher et al., 2008; Kamatani et al., 2011a; Wells et al., 2012; Kamatani et al., 2011b; Zhang et al., 2019; Lertnawapan and Jatuworapruk, 2021; Liu et al., 2022; O'Dell et al., 2022; Hosoya et al., 2016; Kim et al., 2014; Hosoya et al., 2017; Reinders et al., 2009; Becker et al., 2005b; Saag et al., 2022; Mu et al., 2019; Jackson et al., 2012; Kojima et al., 2019; Peng et al., 2023), benzbromarone in 5 studies (Liang et al., 2019; Liu et al., 2022; Yan et al., 2022; Xue et al., 2024; Reinders et al., 2009), topiroxostat in 2 studies (Hosoya et al., 2016; Hosoya et al., 2017), febuxostat 20 mg in 6 studies (Liang et al., 2019; Liu et al., 2022; Yan et al., 2022; Xue et al., 2024; Kamatani et al., 2011c; Lee et al., 2023), febuxostat 40 mg in 19 studies (Xu et al., 2015; Becker et al., 2010; Kamatani et al., 2011a; Wells et al., 2012; Kamatani et al., 2011b; Zhang et al., 2019; Lertnawapan and Jatuworapruk, 2021; O'Dell et al., 2022; Becker et al., 2005a; Kamatani et al., 2011c; Lee et al., 2023; Yamanaka et al., 2018; Afinogenova et al., 2022), febuxostat 80 mg in 14 studies (Becker et al., 2009; Xu et al., 2015; Becker et al., 2010; Schumacher et al., 2008; Wells et al., 2012; Zhang et al., 2019; Lertnawapan and Jatuworapruk, 2021; Becker et al., 2005a; Schumacher et al., 2009; Kim et al., 2014; Becker et al., 2005b; Mu et al., 2019; Peng et al., 2023; Afinogenova et al., 2022), febuxostat 120 mg in 6 studies (Becker et al., 2009; Schumacher et al., 2008; Becker et al., 2005a; Schumacher et al., 2009; Kim et al., 2014; Becker et al., 2005b), allopurinol 100 mg in 3 studies (Lertnawapan and Jatuworapruk, 2021; O'Dell et al., 2022; Yamanaka et al., 2018), allopurinol 200 mg in 3 studies (Zhang et al., 2019; Hosoya et al., 2016; Hosoya et al., 2017), allopurinol 300 mg in 6 studies (Xu et al., 2015; Kamatani et al., 2011a; Zhang et al., 2019; Lertnawapan and Jatuworapruk, 2021; Liu et al., 2022; Kim et al., 2014), and allopurinol 200/300 mg in 3 studies (Becker et al., 2010; Wells et al., 2012; Jackson et al., 2012), benzbromarone 25 mg in 4 studies (Liang et al., 2019; Liu et al., 2022; O'Dell et al., 2022; Yan et al., 2022), and benzbromarone 100 mg in 1 study (Reinders et al., 2009). For outcome indicators, SUA levels were reported in 21 studies (Xu et al., 2015; Kamatani et al., 2011a; Kamatani et al., 2011b; Liang et al., 2019; Zhang et al., 2019; Lertnawapan and Jatuworapruk, 2021; Liu et al., 2022; O'Dell et al., 2022; Yan et al., 2022; Xue et al., 2024; Hosoya et al., 2016; Lee et al., 2023; Kim et al., 2014; Hosoya et al., 2017; Reinders et al., 2009; Saag et al., 2022; Jackson et al., 2012; Kojima et al., 2019; Saag et al., 2016; Kamatani et al., 2011c; Peng et al., 2023), the proportion of individuals achieved target SUA levels in 9 studies (Becker et al., 2009; Becker et al., 2010; Schumacher et al., 2008; Wells et al., 2012; Becker et al., 2005a; Yamanaka et al., 2018; Schumacher et al., 2009; Hosoya et al., 2017; Mu et al., 2019), gout flare in 10 studies (Becker et al., 2009; Xu et al., 2015; Becker et al., 2010; O'Dell et al., 2022; Yamanaka et al., 2018; Becker et al., 2005b; Saag et al., 2022; Jackson et al., 2012; Kojima et al., 2019; Peng et al., 2023), and adverse events in 16 studies (Becker et al., 2009; Xu et al., 2015; Becker et al., 2010; Schumacher et al., 2008; Zhang et al., 2019; O'Dell et al., 2022; Xue et al., 2024; Becker et al., 2005a; Kamatani et al., 2011c; Yamanaka et al., 2018; Reinders et al., 2009; Peng et al., 2023; Afinogenova et al., 2022).

**TABLE 1:** Characteristics of the selected studies.

Author (Year)	Country	Male/Female	Age(m ± SD)	Sample size (n)	Intervention	Course (Weeks)	Outcomes
Becker et al. (2009)	United States	-	51.4 ± 11.95	466	Feb_80 mg	24	②③④
		-	50.9 ± 11.57	199	Feb_120 mg		
		-	51.0 ± 11.30	63	All_300 mg		
Xu et al. (2015)	China	146/12	48.2 ± 12.0	158	Feb_80 mg	24	①③④
		158/2	45.5 ± 11.9	160	Feb_40 mg		
		149/10	46.6 ± 10.7	159	All_300 mg		
Becker et al. (2010)	United States	722/35	52.5 ± 11.68	757	Feb_40 mg	24	②④
		710/46	53.0 ± 11.79	756	Feb_80 mg		
		709/47	52.9 ± 11.73	755	All_200_300 mg		
Schumacher et al. (2008)	United States	251/16	51 ± 12	161	Feb_80 mg	28	②④
		255/14	51 ± 12	188	Feb_120 mg		
		125/9	54 ± 13	83	Feb_240 mg		
		250/18	52 ± 12	208	All_300 mg		
		123/11	52 ± 12	99	Placebo		
Kamatani et al. (2011a)	Japan	10/0	56 ± 8.2	10	Feb_40 mg	16	①
		9/0	53.3 ± 11.0	9	Feb_60 mg		
		19/0	51.3 ± 12.0	10	All_300 mg		
Wells et al. (2012)	United States	-	-	703	Feb_40 mg	24	②③
		-	-	696	Feb_80 mg		
		-	-	692	All_200_300 mg		
Kamatani et al. (2011b)	Japan	119/3	51.6 ± 13.1	122	Feb_40 mg	8	①
		118/2	52.6 ± 14.0	122	All_200 mg		
Liang et al. (2019)	China	-	52.42 ± 11.73	105	Feb_20 mg	12	①③
		-	50.27 ± 14.15	109	Ben_25 mg		
Zhang et al. (2019)	China	180/1	46.5 ± 11.9	181	Feb_40 mg	24	①④
		184/4	47.2 ± 12.9	188	Feb_80 mg		
		182/2	48.3 ± 13.1	184	All_300 mg		
Lertnawapan and Jatuworapruk. (2021)	Thailand	89/31	68.51 ± 13.74	55	Feb_40 mg	27	①
		105/15	62.04 ± 13.47	59	Feb_80 mg		
		39/16	69.78 ± 14.81	120	All_100 mg		
		46/13	62.53 ± 13.73	120	All_300 mg		
Liu et al. (2022)	China	46/4	47.68 ± 15.88	23	Feb_20 mg	4	①
		23/0	48.30 ± 15.76	50	Ben_25 mg		
O'Dell et al. (2022)	United States	465/7	62.9 ± 11.8	472	Feb_40 mg	24	①③④
		460/18	61.3 ± 12.9	468	All_100 mg		
Yan et al. (2022)	China	98/0	43.89 ± 13.10	98	Feb_20 mg	12	①③
		98/0	43.29 ± 12.22	98	Ben_25 mg		
Xue et al. (2024)	China	125/0	39.9 ± 2.71	125	Feb_20 mg	12	①③④
		125/0	39.1 ± 3.29	125	Feb_20 mg_Ben_25 mg		
Hosoya et al. (2016)	Japan	104/1	53.7 ± 11.9	105	All_200 mg	16	①
		97/1	52.3 ± 10.9	98	Top_120 mg		
Becker et al. (2005a)	United States	33/4	52.2 ± 14.0	37	Feb_40 mg	4	②③④
		38/2	55.2 ± 13.1	40	Feb_80 mg		
		33/5	56.2 ± 10.8	38	Feb_120 mg		
		32/6	52.4 ± 12.6	38	Placebo		
Kamatani et al. (2011c)	Japan	35/0	50.9 ± 14.0	35	Feb_20 mg	8	①④
		34/0	43.4 ± 13.6	34	Feb_40 mg		
		33/0	48.2 ± 13.4	33	Placebo		
Li et al. (2023)	Korea	89/5	56.5 ± 16.8	94	Feb_20 mg	12	①
		130/3	50.8 ± 15.8	133	Feb_40 mg		
Yamanaka et al. (2018)	Japan	-	47.4 ± 10.5	90	Feb_10_40 mg	12	②③④
		-	46.4 ± 12.7	42	Feb_40 mg		
Schumacher et al. (2009)	United States	-	-	10	Feb_40 mg	28	②③
		-	-	58	Feb_80 mg		
		-	-	17	Feb_120 mg		
Huang et al. (2014)	China	167/5	46.42 ± 10.90	172	Feb_40 mg	28	①③④
		169/3	47.4 ± 11.18	172	Feb_80 mg		
		168/4	46.17 ± 11.56	172	All_300 mg		
Kim et al. (2014)	Korea	-	49.6 ± 11.9	35	Feb_40 mg	4	①
		-	49.1 ± 12.4	35	Feb_80 mg		
		-	51.2 ± 9.9	36	Feb_120 mg		
		-	48.3 ± 11.8	36	All_300 mg		
		-	51.8 ± 12.4	37	Placebo		
Hosoya et al. (2017)	Japan	38/1	50.7 ± 8.4	39	Top_120 mg	16	①
		38/2	53.2 ± 7.9	40	Top_160 mg		
		38/0	52.3 ± 8.4	39	All_200 mg		
		39/0	49.6 ± 8.1	38	Placebo		
Reinders et al. (2009)	Netherlands	26/6	58.6 ± 12.3	31	All_300 mg	8	①③④
		21/4	59.6 ± 11.3	25	Ben_100 mg		
Becker et al. (2005b)	United States	243/13	51.8 ± 11.7	159	Feb_80 mg	52	②③④
		243/8	52.0 ± 12.1	145	Feb_120 mg		
		243/10	51.6 ± 12.6	178	All_300 mg		
Saag et al. (2022)	United States	-	-	2,964	Feb_40 mg	24	①③
		-	-	2,992	All_300 mg		
Mu et al. (2019)	China	122/6	46.1 ± 11.4	128	Feb_80 mg	24	②
		102/10	47.3 ± 11.9	112	Feb_40 mg		
		122/8	46.8 ± 11.3	130	All_300 mg		
Jackson et al. (2012)	United States	104/11	70.8 ± 5.19	102	Feb_80 mg	24	①③④
		109/19	71.2 ± 5.22	90	Feb_40 mg		
		108/23	70.1 ± 4.59	99	All_200_300 mg		
Kojima et al. (2019)	Japan	371/166	75.4 ± 6.7	537	Feb_40 mg	24	①③④
		368/165	76.0 ± 6.5	533	All_100 mg		
Saag et al. (2016)	United States	25/7	67.3 ± 11.11	32	Feb_60 mg	24	①④
		26/6	63.6 ± 8.15	32	Feb_40_80 mg		
		26/6	66.3 ± 12.05	32	Placebo		

Note: m, mean; SD, standard deviation.①: Serum uric acid; ②: The proportion of people with SUA, levels <6.0 mg; ③: Gout flare ④: Safety outcome.

### 3.3 ROB assessment of the included studies

**FIGURE 2:**
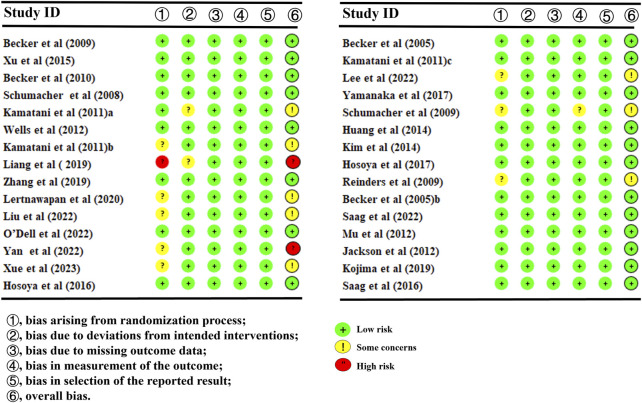
Overall risk of bias of the included studies. For each domain evaluated, the percentages of low, medium, and high risk of bias were as follows: randomization sequence generation (70%, 26.7%, and 3.3%).

The results of the ROB assessment are available in Figure 2. Among the included studies, 23 studies (Becker et al., 2009; Xu et al., 2015; Becker et al., 2010; Schumacher et al., 2008; Wells et al., 2012; Zhang et al., 2019; Liu et al., 2022; O'Dell et al., 2022; Yan et al., 2022; Xue et al., 2024; Hosoya et al., 2016; Becker et al., 2005a; Kamatani et al., 2011c; Yamanaka et al., 2018; Kim et al., 2014; Hosoya et al., 2017; Becker et al., 2005b; Saag et al., 2022; Mu et al., 2019; Jackson et al., 2012; Kojima et al., 2019; Saag et al., 2016; Peng et al., 2023) were grouped according to a random number table. The remaining eight studies only mentioned random or did not describe the specific grouping. Data integrity was favorable in all studies. Nevertheless, allocation concealment, blinded interventions, and other sources of ROB were not mentioned. The overall ROB of these included studies was average.

### 3.4 NMA results

#### 3.4.1 Primary outcomes

##### 3.4.1.1 SUA levels

**FIGURE 3:**
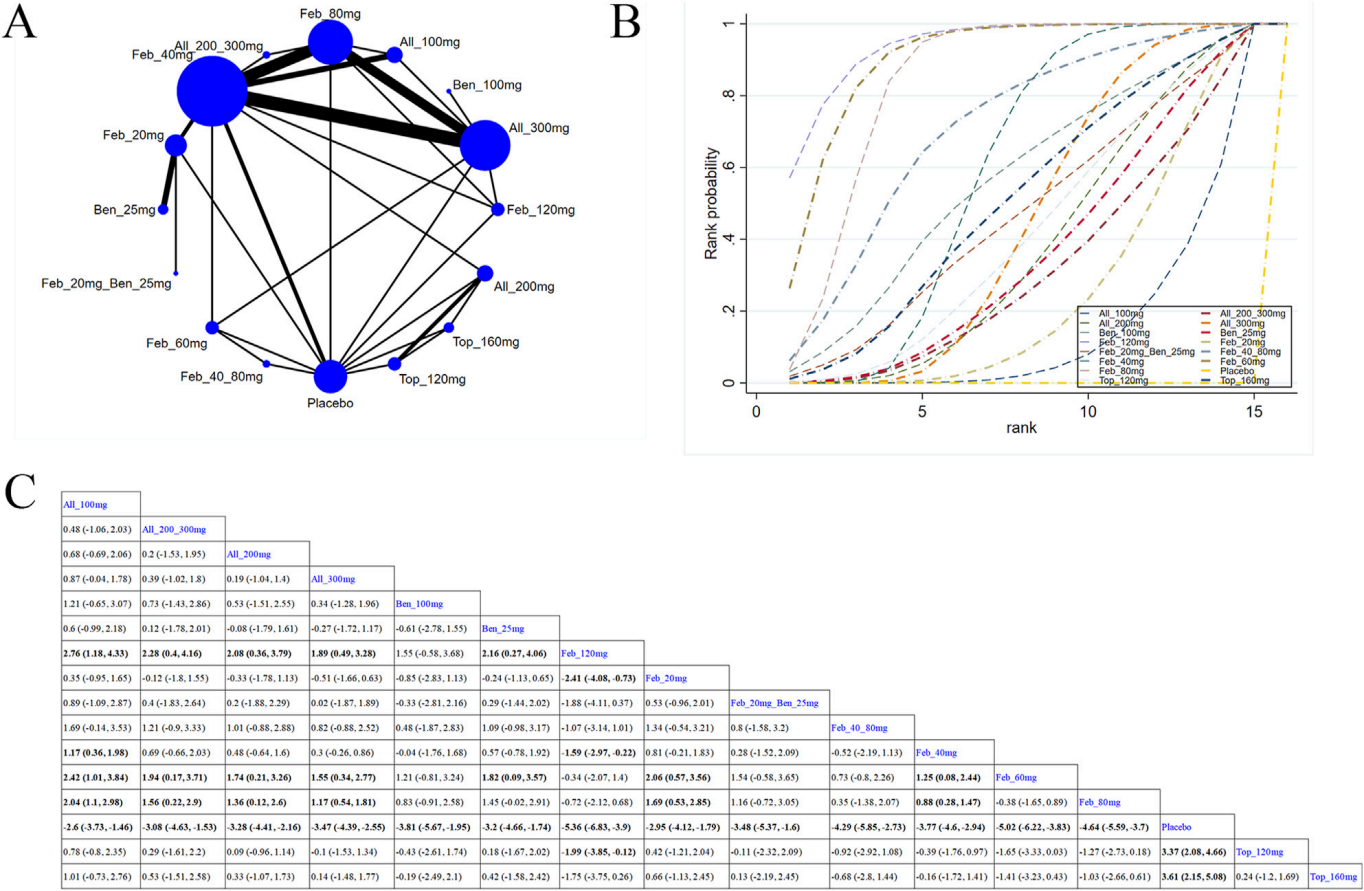
Network plot and results of network meta-analysis. **(A)** Network plot for the primary outcome. **(B)** Cumulative ranking plots for the primary outcome. **(C)** Relative effects of different ULTs on the primary outcome. Notes: estimates are presented as mean differences (MDs) and 95% confidence intervals (CIs). Comparisons between treatments should be interpreted from left to right. The estimate for supplementation effectiveness is positioned at the intersection of the column and row defining the respective supplementations.

30 studies reported SUA levels, as well as the number of individuals whose SUA levels reached <6.0 mg/L (Pedder, 2021; Dusseldorp et al., 2014; Wickham, 2011; Becker et al., 2009; Xu et al., 2015; Becker et al., 2010; Schumacher et al., 2008; Kamatani et al., 2011a; Wells et al., 2012; Kamatani et al., 2011b; Liang et al., 2019; Zhang et al., 2019; Lertnawapan and Jatuworapruk, 2021; Liu et al., 2022; O'Dell et al., 2022; Yan et al., 2022; Xue et al., 2024; Hosoya et al., 2016; Becker et al., 2005a; Kamatani et al., 2011c; Lee et al., 2023; Yamanaka et al., 2018; Schumacher et al., 2009; Kim et al., 2014; Hosoya et al., 2017; Reinders et al., 2009; Peng et al., 2023; Afinogenova et al., 2022). Specifically, 21 (Becker et al., 2010; Wells et al., 2012; Liang et al., 2019; Zhang et al., 2019; Lertnawapan and Jatuworapruk, 2021; Liu et al., 2022; O'Dell et al., 2022; Yan et al., 2022; Xue et al., 2024; Hosoya et al., 2016; Becker et al., 2005a; Lee et al., 2023; Yamanaka et al., 2018; Hosoya et al., 2017; Reinders et al., 2009; Becker et al., 2005b; Mu et al., 2019; Kojima et al., 2019; Saag et al., 2016; Peng et al., 2023; Afinogenova et al., 2022) studies reported SUA levels (Figure 3A). The NMA findings are displayed in Figure 3B. All ULTs resulted in noticeably lower SUA levels than placebo/non-ULT (Figure 3C). Febuxostat 120 mg outperformed other treatment groups (such as the allopurinol groups, other dose groups of febuxostat, the benzbromarone groups, and the topixostat groups) in reducing SUA levels. It notably reduced SUA levels compared to each dose group of allopurinol and benzbromarone 25 mg (MD = 2.16, 95% CI [0.27, 4.06]), febuxostat 40 mg (MD = −1.59, 95% CI [-2.97, −0.22]) and 20 mg (MD = −2.41, 95% CI [-4.08, −0.73]), topiroxostat 120 mg (MD = −1.99, 95% CI [-3.85, −1.02]), and placebo (MD = −5.36, 95% CI [-6.83, −3.9]). Febuxostat 80 mg was more effective than allopurinol and febuxostat 40 mg (MD = 0.88, 95% CI [0.28, 1.47]) and febuxostat 20 mg (MD = 2.06, 95% CI [0.57, 3.56]). Febuxostat 40 mg was notably superior to allopurinol 100 mg (MD = 1.25, 95% CI [0.08, 2.44]). According to the SUCRA, febuxostat 120 mg was the best treatment to decrease SUA levels (SUCRA = 94%) (Supplementary Table S1). Nine studies (Xu et al., 2015; Schumacher et al., 2008; Kamatani et al., 2011a; Kamatani et al., 2011b; Kamatani et al., 2011c; Schumacher et al., 2009; Saag et al., 2022; Jackson et al., 2012; Peng et al., 2023) reported the proportion of individuals with SUA levels <6.0 mg. According to the SUCRA, febuxostat 240 mg was the best treatment to decrease SUA levels (SUCRA = 99.6%) (Supplementary Table S2). The evidence quality of this outcome measure was evaluated by the GRADE system (Supplementary Figure S3).

##### 3.4.1.2 Dose-response analysis of the association between circulating ULTs and gout patients

**FIGURE 4:**
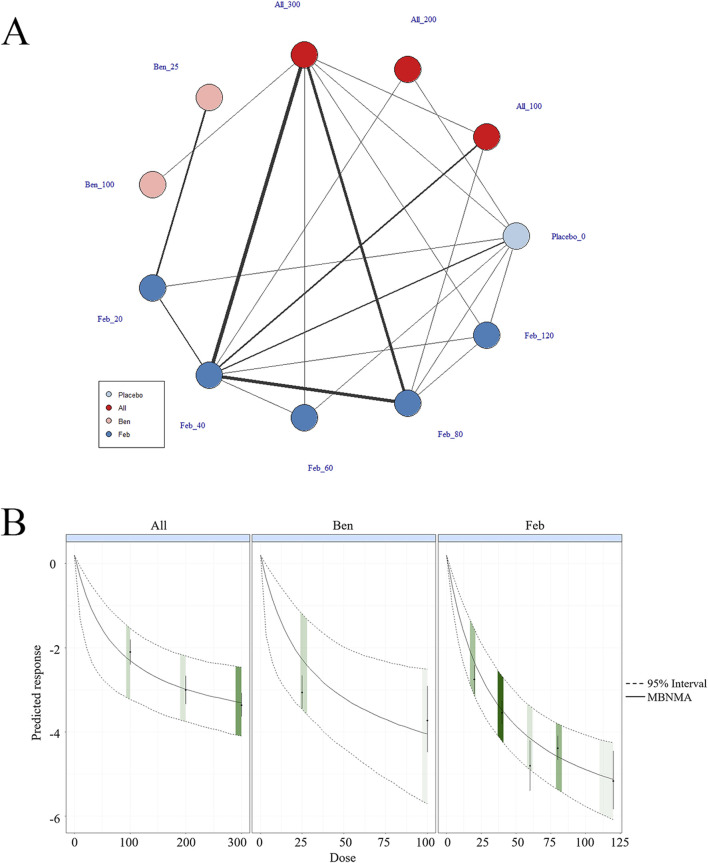
Network plot and results of network meta-analysis. **(A)** Network map of evidence for eligible comparisons. The numbers denote distinct intervention measures. Blue node size represents the participant count for each intervention type, and line thickness between interventions indicates the volume of comparative studies. **(B)** Dose-response association between ULT drug doses and serum uric acid levels.

According to the results of different ULTs, dose-response analysis on the relationship between dose and SUA levels was executed to identify the optimal therapeutic dose for different ULTs in gout patients. In Figure 4, the dose-response curves for each intervention examined in this study are displayed. An inverted dose-response relationship was observed, where increased doses of ULTs led to reductions in SUA levels. It was worth noting that the dose-response association was specific to each intervention. The doses of ULTs were positively linked to SUA levels. As the dosage of the drug increased, SUA levels decreased (Figure 4).

##### 3.4.1.3 Subgroup analysis

###### 3.4.1.3.1 Treatment for <12 weeks

**FIGURE 5:**
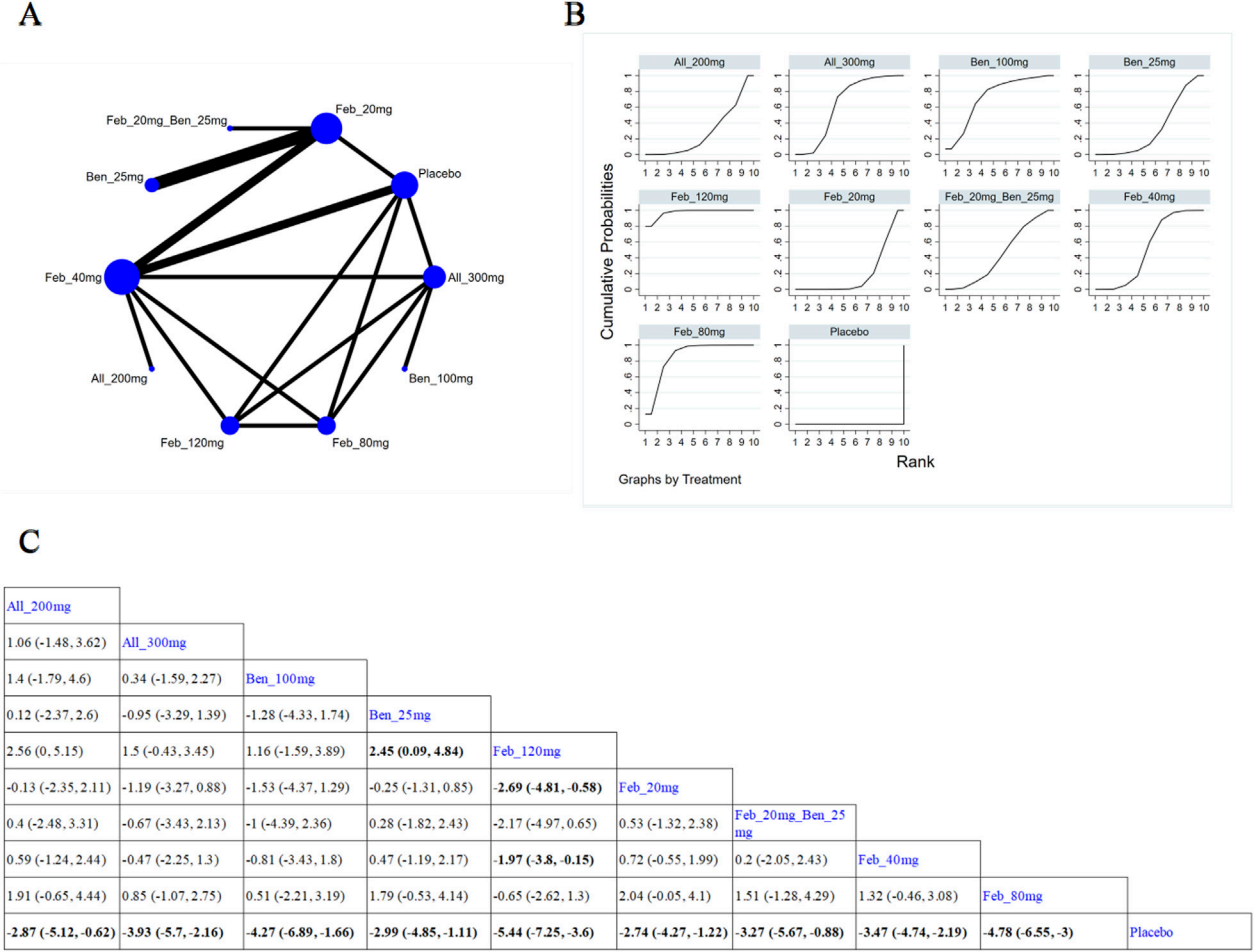
Network plot and results of network meta-analysis. **(A)** Network plot for treatment for < 12 weeks. **(B)** Cumulative ranking plots for treatment for < 12 weeks. **(C)** Relative effects of different ULTs on gout flare. Notes: estimates are presented as mean differences (MDs) and 95% confidence intervals (CIs). Comparisons between treatments should be interpreted from left to right. The estimate for supplementation effectiveness is positioned at the intersection of the column and row defining the respective supplementations.

Nine (Liang et al., 2019; Zhang et al., 2019; Liu et al., 2022; Xue et al., 2024; Hosoya et al., 2016; Lee et al., 2023; Yamanaka et al., 2018; Hosoya et al., 2017; Becker et al., 2005b) studies reported SUA levels within 12-week treatment (Figure 5A). The NMA findings are illustrated in Figure 5B. All ULTs resulted in markedly lower SUA levels than placebo/non-ULT (Figure 5C). Febuxostat 120 mg was superior to other treatment groups (like the allopurinol groups, other dose groups of febuxostat, the benzbromarone groups, and the topixostat groups). It notably reduced SUA levels compared to benzbromarone 25 mg (MD = 2.45, 95% CI [0.09, 4.84]), febuxostat 40 mg (MD = −1.97, 95% CI [-3.8, −0.15]) and 20 mg (MD = −2.69, 95% CI [-4.81, −0.58]), and placebo (MD = −5.44, 95% CI [-7.25, −3.6]). According to the SUCRA, febuxostat 120 mg was the best treatment to decrease SUA levels (SUCRA = 97.3%) (Supplementary Table S4).

###### 3.4.1.3.2 Treatment for ≥12 weeks

**FIGURE 6:**
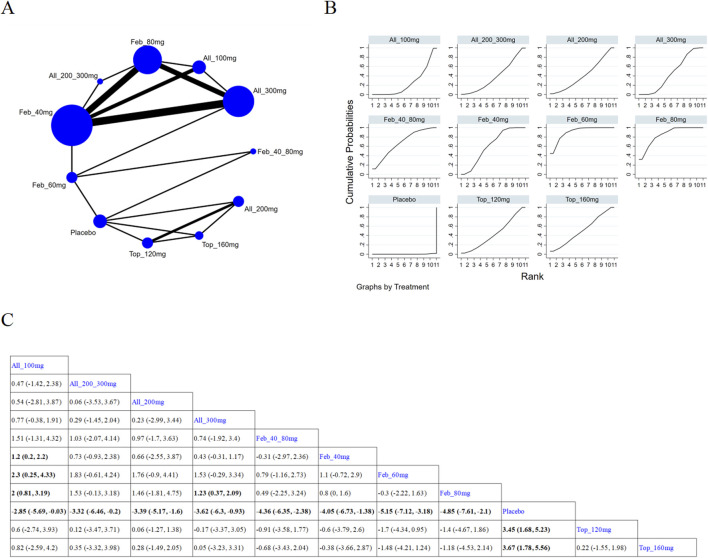
Network plot and results of network meta-analysis. **(A)** Network plot for treatment for ≥ 12 weeks. **(B)** Cumulative ranking plots for treatment for ≥ 12 weeks. **(C)** Relative effects of different ULTs on gout flare. Notes: estimates are presented as mean differences (MDs) and 95% confidence intervals (CIs). Comparisons between treatments should be interpreted from left to right. The estimate for supplementation effectiveness is positioned at the intersection of the column and row defining the respective supplementations.

12 (Becker et al., 2010; Wells et al., 2012; Lertnawapan and Jatuworapruk, 2021; Liu et al., 2022; Yan et al., 2022; Becker et al., 2005a; Kim et al., 2014; Reinders et al., 2009; Mu et al., 2019; Kojima et al., 2019; Saag et al., 2016; Afinogenova et al., 2022) studies reported SUA levels after 12-week treatment (Figure 6A). The NMA findings are presented in Figure 6B. All ULTs resulted in remarkably lower SUA levels than placebo/non-ULT (Figure 6C). Febuxostat 120 mg was superior to other treatment groups (like the allopurinol groups, other dose groups of febuxostat, the benzbromarone groups, and the topixostat groups). It notably reduced SUA levels compared to each dose group of allopurinol and benzbromarone 25 mg (MD = 2.16, 95% CI [0.27, 4.06]), febuxostat 40 mg (MD = −1.59,95% CI [-2.97, −0.22]) and 20 mg (MD = −2.41, 95% CI [-4.08, −0.73]), topiroxostat 120 mg (MD = −1.99, 95% CI [-3.85, −1.02]), and placebo (MD = −5.36, 95% CI [-6.83, −3.9]). According to the SUCRA, febuxostat 60 mg was the best treatment to decrease SUA levels (SUCRA = 90.4%) (Supplementary Table S5).

#### 3.4.2 Gout flare

**FIGURE 7:**
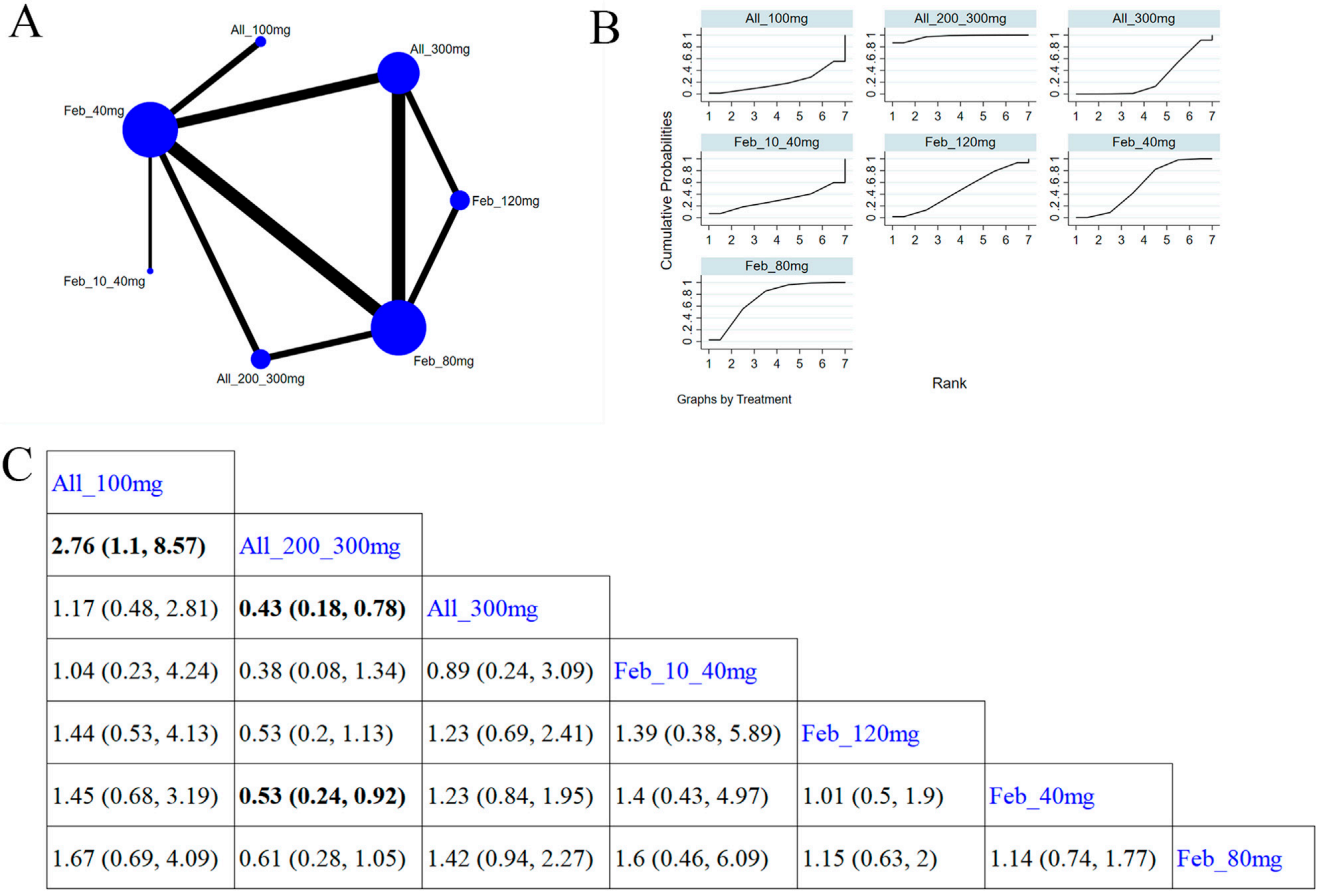
Network plot and results of network meta-analysis. **(A)** Network plot for gout flare. **(B)** Cumulative ranking plots for gout flare. **(C)** Relative effects of different ULTs on gout flare. Notes: estimates are presented as mean differences (MDs) and 95% confidence intervals (CIs). Comparisons between treatments should be interpreted from left to right. The estimate for supplementation effectiveness is positioned at the intersection of the column and row defining the respective supplementations.

11 studies (Pedder, 2021; Dusseldorp et al., 2014; Wickham, 2011; Becker et al., 2009; Zhang et al., 2019; Becker et al., 2005a; Lee et al., 2023; Kim et al., 2014; Hosoya et al., 2017; Becker et al., 2005b; Saag et al., 2022) reported gout flare (Figure 7A). The NMA findings are illustrated in Figure 7B. Allopurinol 200/300 mg obviously reduced patients’ gout flare compared to allopurinol 100 mg (MD = 2.76, 95% CI [1.1, 8.57]), allopurinol 300 mg (MD = 0.43, 95% CI [0.18, 0.78]), and febuxostat 40 mg (MD = 0.53, 95% CI [0.24, 0.92]) (Figure 7C). Febuxostat 80 mg was superior to febuxostat 40 mg in reducing gout flare (MD = 1.14,95% CI [0.74, 1.77]). According to the SUCRA, allopurinol 200/300 mg was the best choice to reduce patients’ gout flare (SUCRA = 96.9%) (Supplementary Table S6).

#### 3.4.3 Safety outcome

##### 3.4.3.1 Cardiovascular events

**FIGURE 8:**
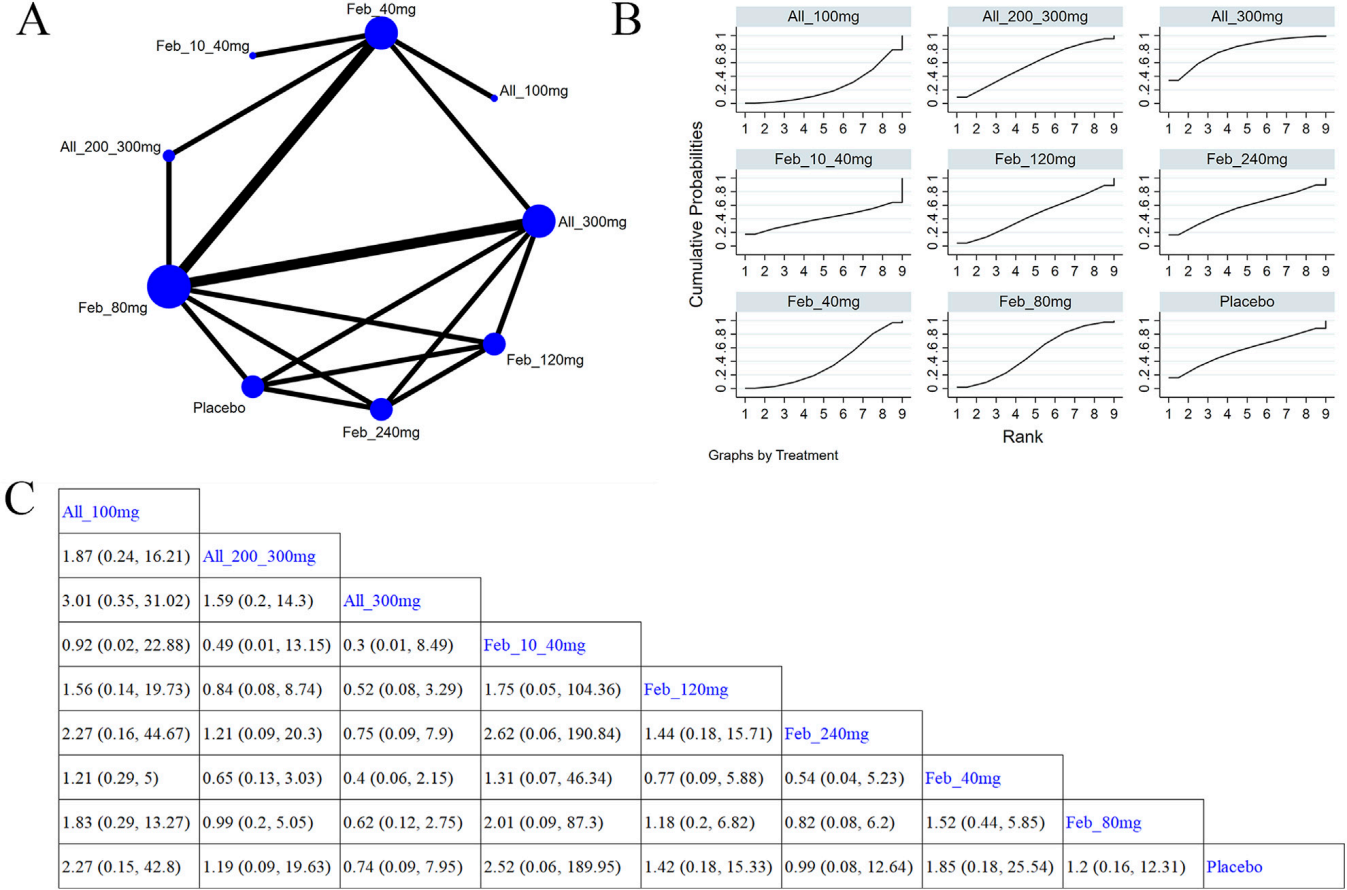
Network plot and results of network meta-analysis. **(A)** Network map of evidence for eligible comparisons. The numbers denote distinct intervention measures. Blue node size represents the participant count for each intervention type, and line thickness between interventions indicates the volume of comparative studies. **(B)** Cumulative ranking plots for cardiovascular events. The area under the curve indicates the efficacy of different treatment measures. A larger area under the curve indicates better efficacy. **(C)** League table for each intervention based on the SUCRA values.

Five studies (Xu et al., 2015; Becker et al., 2010; Schumacher et al., 2008; O'Dell et al., 2022; Yamanaka et al., 2018) investigated cardiovascular events (Figure 8A). The NMA findings are displayed in Figure 8B. No notable difference was noted in cardiovascular events among different ULTs (Figure 8C). According to the SUCRA, allopurinol 300 mg was the best choice to reduce the occurrence of cardiovascular events (SUCRA = 73.5%) (Supplementary Table S7).

##### 3.5.3.2 Abnormal liver function

**FIGURE 9:**
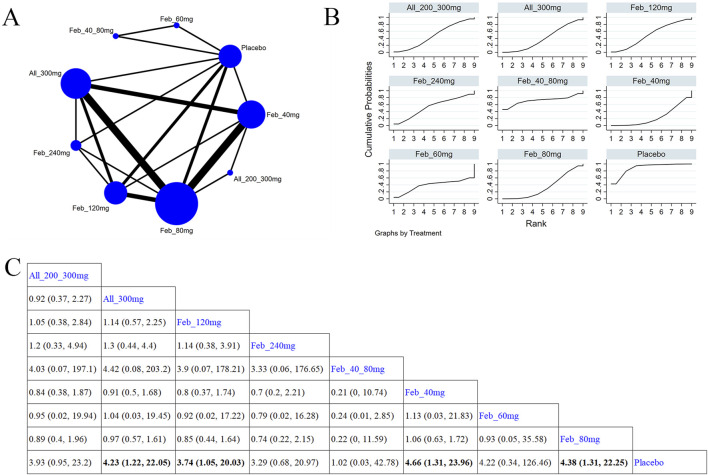
Network plot and results of network meta-analysis. **(A)** Network map of evidence for eligible comparisons. The numbers denote distinct intervention measures. Blue node size represents the participant count for each intervention type, and line thickness between interventions indicates the volume of comparative studies. **(B)** Cumulative ranking plots for abnormal liver function. The area under the curve indicates the efficacy of different treatment measures. A larger area under the curve indicates better efficacy. **(C)** League table for each intervention based on the SUCRA values.

Eight studies (Xu et al., 2015; Becker et al., 2010; Schumacher et al., 2008; Zhang et al., 2019; Becker et al., 2005a; Reinders et al., 2009; Saag et al., 2016; Peng et al., 2023) examined abnormal liver function (Figure 9A). The NMA findings are presented in Figure 9B. Compared with allopurinol 300 mg, febuxostat 120 mg (MD = 4.23, 95% CI [1.22, 22.05]), 80 mg (MD = 3.74, 95% CI [1.05, 20.03]), and 40 mg (MD = 4.38, 95% CI [1.31, 22.25]), placebo (MD = 4.66, 95% CI [1.31, 23.96]) remarkably improved patients’ liver function (Figure 9C). No notable difference was observed in abnormal liver function between febuxostat and allopurinol. According to the SUCRA, placebo was the best choice to reduce the occurrence of abnormal liver function (SUCRA = 90.4%) (Supplementary Table S8).

##### 3.5.3.3 Abnormal renal function

**FIGURE 10:**
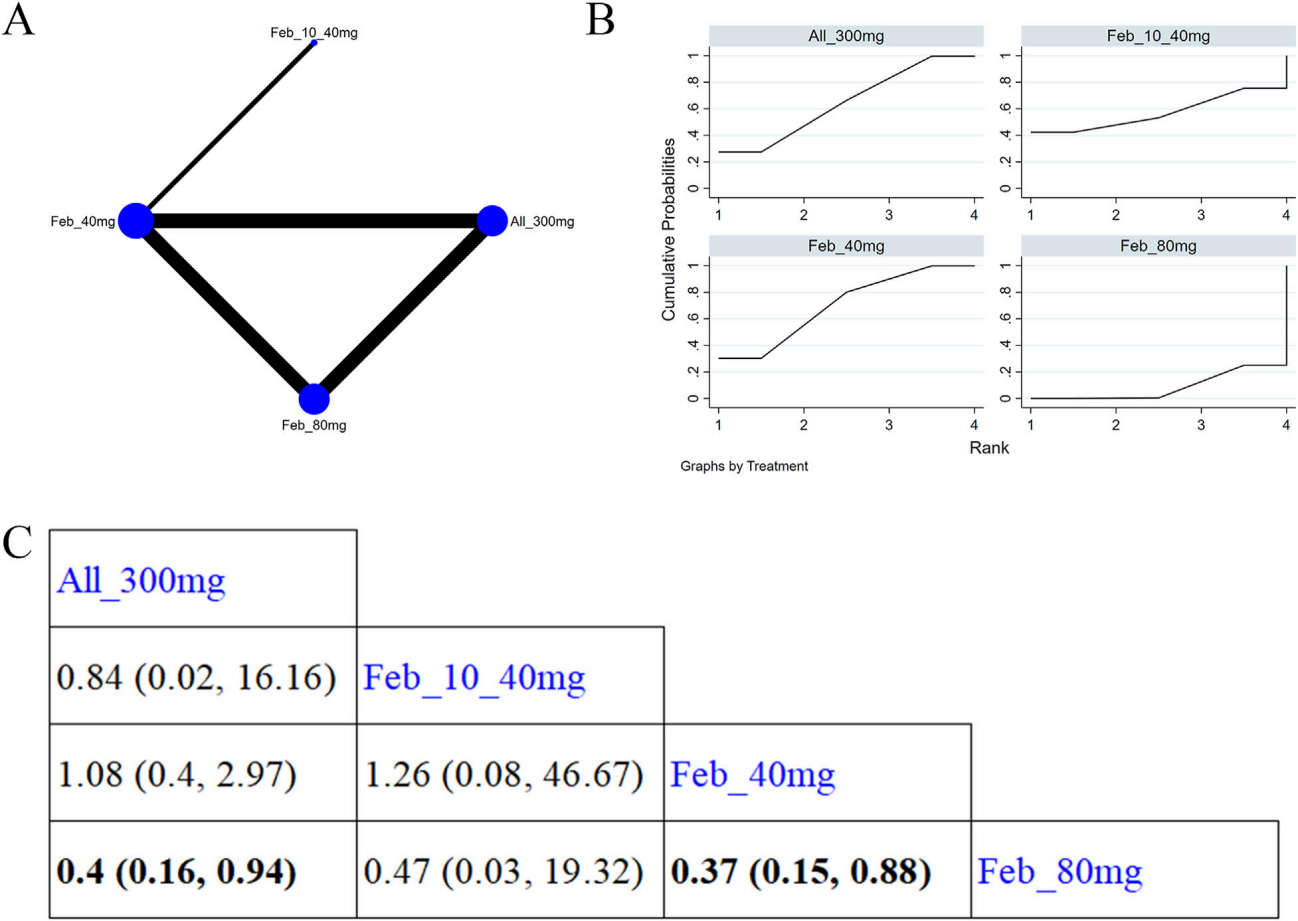
Network plot and results of network meta-analysis. **(A)** Network map of evidence for eligible comparisons. The numbers denote distinct intervention measures. Blue node size represents the participant count for each intervention type, and line thickness between interventions indicates the volume of comparative studies. **(B)** Cumulative ranking plots for abnormal renal function. The area under the curve indicates the efficacy of different treatment measures. A larger area under the curve indicates better efficacy. **(C)** League table for each intervention based on the SUCRA values.

Four studies (Xu et al., 2015; Zhang et al., 2019; Yamanaka et al., 2018; Peng et al., 2023) reported renal function (Figure 10A). Figure 10B illustrates the NMA findings. Compared with febuxostat 80 mg, allopurinol 300 mg (MD = 0.4, 95% CI [0.16, 0.94]) markedly improved patients’ renal function (Figure 10C). Compared to febuxostat 80 mg, febuxostat 40 mg (MD = 0.37, 95% CI [0.15, 0.88]) noticeably enhanced patients’ renal function (Figure 10C). According to the SUCRA, allopurinol 300 mg was the best choice to reduce the occurrence of abnormal renal function (SUCRA = 65.4%) (Supplementary Table S9).

##### 3.5.3.4 Muscle and connective tissue

**FIGURE 11:**
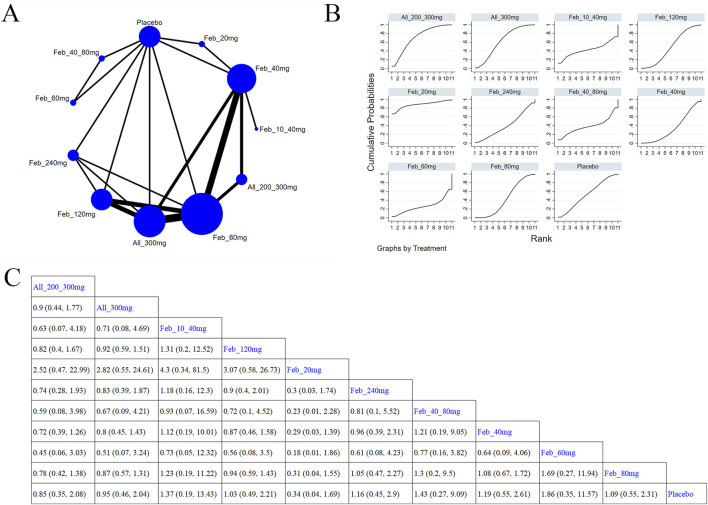
Network plot and results of network meta-analysis. **(A)** Network plot for muscle and connective tissue. **(B)** Cumulative ranking plots for muscle and connective tissue. **(C)** Relative effects of different ULTs on muscle and connective tissue. Notes: estimates are presented as mean differences (MDs) and 95% confidence intervals (CIs). Comparisons between treatments should be interpreted from left to right. The estimate for supplementation effectiveness is positioned at the intersection of the column and row defining the respective supplementations.

Eight studies (Becker et al., 2009; Xu et al., 2015; Becker et al., 2010; Schumacher et al., 2008; Zhang et al., 2019; Becker et al., 2005b; Jackson et al., 2012; Saag et al., 2016) reported muscle and connective tissue (Figure 11A). The NMA findings are presented in Figure 11B. Compared with placebo, febuxostat 20 mg (MD = 0.34, 95% CI [0.04, 1.69]) evidently reduced the occurrence of muscle and connective tissue diseases (Figure 11C). According to the SUCRA, febuxostat 20 mg was the best choice to reduce events for muscle and connective tissues (SUCRA = 89.8%) (Supplementary Table S10).

##### 3.5.3.5 Serious AEs

**FIGURE 12:**
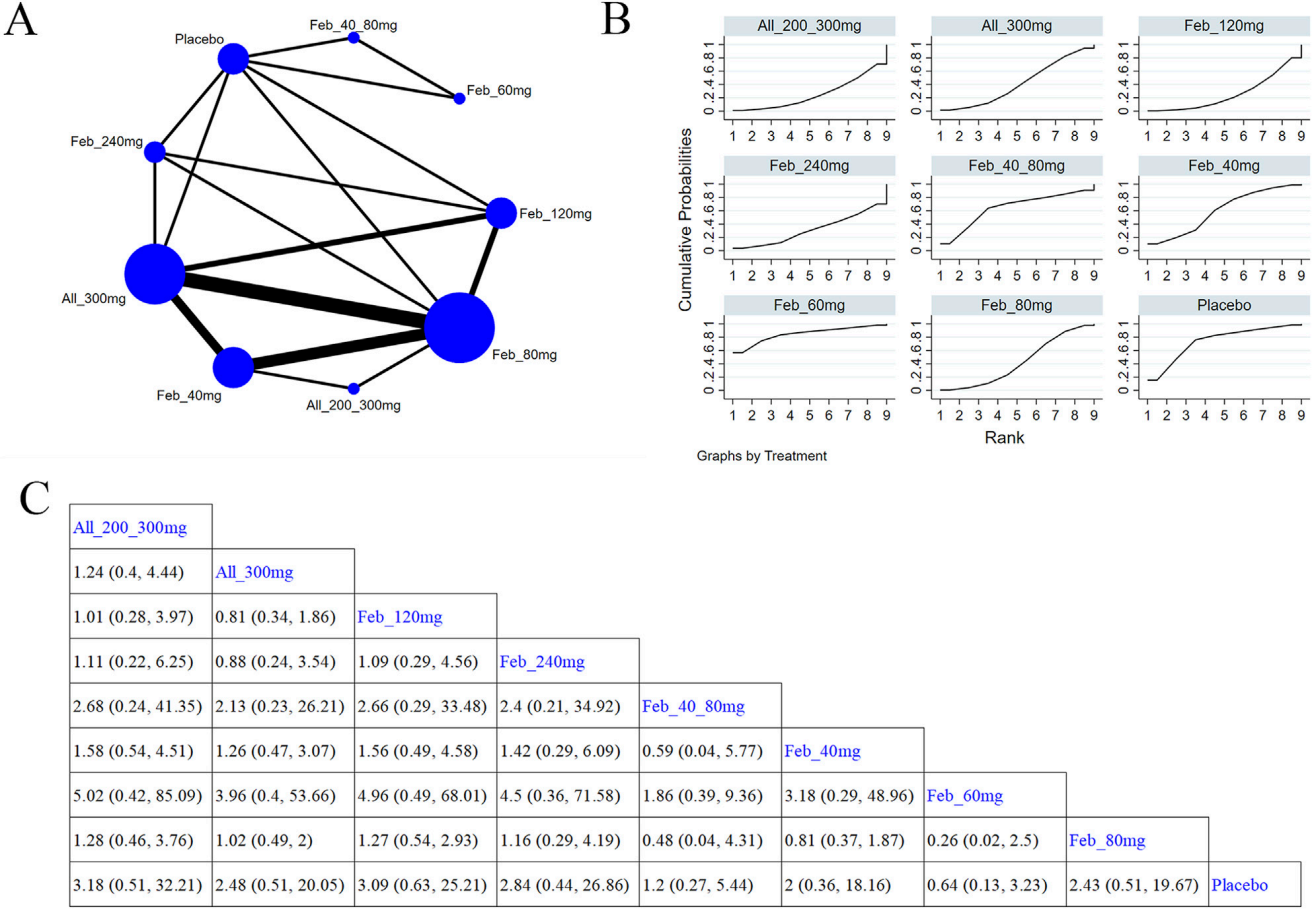
Network plot and results of network meta-analysis. **(A)** Network plot for serious adverse events. **(B)** Cumulative ranking plots for serious adverse events. **(C)** Relative effects of different ULTs on serious adverse events. Notes: estimates are presented as mean differences (MDs) and 95% confidence intervals (CIs). Comparisons between treatments should be interpreted from left to right. The estimate for supplementation effectiveness is positioned at the intersection of the column and row defining the respective supplementations.

Eight studies (Xu et al., 2015; Becker et al., 2010; Schumacher et al., 2008; Wells et al., 2012; Zhang et al., 2019; Hosoya et al., 2016; Reinders et al., 2009; Saag et al., 2016; Peng et al., 2023) explored serious adverse events (Figure 12A). The NMA findings are shown in Figure 12B. Compared with placebo, febuxostat 60 mg and Feb_40_80 mg (MD = 0.64, 95% CI [0.13, 3.23]) markedly reduced the occurrence of heart events (Figure 12C). According to the SUCRA, febuxostat 60 mg was the best choice to reduce the occurrence of serious AEs (SUCRA = 85.1%) (Supplementary Table S11).

#### 3.4.4 Heterogeneity analysis

We conducted an exploration of local heterogeneity for the primary outcome measure, SUA levels. Based on our analysis results, when comparing All 100 mg with All 300 mg, February 40 mg, and February 40 mg, Feb All 300 mg was compared with 40 mg and February 80 mg, and February 40 mg vs. February 20 mg showed significant heterogeneity. The results are presented as follows (Supplementary Table S12).

## 4 Discussion

A comprehensive search of relevant publications is conducted. The NMA analyzes all available evidence from 30 studies to compare the efficacy and AEs of ULTs in treating gout patients. This meta-analysis distinguishes itself from previous studies by specifically focusing on the impact of dosages on efficacy and safety. The results provide a theoretical basis and guidance for clinical drug treatment. The findings highlight the advantages of febuxostat in reducing SUA levels to below 6.0 mg/dL. In detail, our findings indicate that febuxostat demonstrates greater efficacy in reducing SUA levels at a daily dose of 120 mg. ULTs exhibit a linear relationship with dose, with SUA levels decreasing as the dose increases. The incidence of gout is lower with allopurinol 200/300 mg. Allopurinol 300 mg is tied to lower incidences of cardiovascular and renal abnormalities, febuxostat 20 mg with fewer muscle and connective tissue events, and febuxostat 60 mg with fewer serious AEs. Moreover, the incidence of AEs increases with increasing doses.

According to the 2020 ACR gout guidelines (Wu et al., 2024), it is strongly advised to start ULT in patients who have one or more clinically evident tophi, radiographic evidence of gouty bone erosion, or experience two or more gout flares per year. In the ACR gout guidelines (Wu et al., 2024), the treat-to-target strategy involves achieving an SUA level of <6 mg/dL, reducing the frequency of gout flares, and further alleviating tophus after 2 years. Prior systematic reviews and meta-analyses have indicated that drugs such as febuxostat, allopurinol, benzbromarone, and topirxostat reduce SUA levels (Wu et al., 2024; Lee and Song, 2022a; Lee and Song, 2022b). This finding is consistent with our results. Hyperuricemia is a key factor in both the onset and recurrence of gout. Maintaining a sustained reduction in SUA levels through ULT is crucial for the long-term management of gout, as it helps to dissolve MSU crystals, reduce gout flares, and eliminate tophi. In this study, febuxostat 120 mg is found to be superior to other treatment groups, and noticeably reduces SUA levels than each dose group of allopurinol and benzbromarone 25 mg, febuxostat 40 mg, and febuxostat 20 mg. Topiroxostat 120 mg and febuxostat 80 mg are notably effective than each dose group of allopurinol and febuxostat 40 mg and febuxostat 20 mg. Febuxostat 40 mg markedly outperforms allopurinol 100 mg in reducing SUA levels. Febuxostat, as a potential xanthine oxidase (XO) inhibitor, reduces uric acid production by inhibiting XO. Moreover, it can reduce superoxide anion. Febuxostat can be metabolized by the liver without adjustment of the dose level. Therefore, it can be used for treating allopurinol allergy or chronic renal insufficiency. Febuxostat demonstrates greater efficacy than allopurinol in inhibiting XO and reducing SUA levels. It does not noticeably affect other enzymes involved in purine and pyrimidine metabolism. Febuxostat may lead to a moderate increase in SUA concentrations, ranging from 0.36 to 0.42 mmol/L, and can prevent the onset of acute gout.

In clinical practice, allopurinol is commonly used as the first-line treatment for inhibiting uric acid synthesis (Peng et al., 2023). This meta-analysis suggests that allopurinol 200/300 mg is the best choice to reduce gout flare in patients. It is better than 100 mg and 300 mg doses. Additionally, allopurinol 200/300 mg is also more effective than each dose of febuxostat. Febuxostat 80 mg is superior to febuxostat 40 mg in reducing gout flare in patients. Allopurinol and its metabolite oxopurinol inhibit the decrease of XO and prevent the metabolism of hypoxanthine and xanthine to uric acid, thereby reducing the synthesis of uric acid. Allopurinol has a single target of action and plays a crucial role in long-term uric acid reduction by stabilizing persistent SUA levels and decreasing the frequency of gout attacks. Notably, this study finds that febuxostat combined with allopurinol may result in a lower attack rate by reducing SUA levels. This finding provides a rationale for future gout treatment.

Most patients need to continue using ULTs for an extended period or even for life. However, prolonged use of these medications can lead to certain side effects. Hence, finding safe and effective strategies for preventing and treating hyperuricemia is crucial for both clinical practice and public health. After ingestion, allopurinol is metabolized into the active form hydroxypurinol in the liver and then excreted through the kidneys. In patients with renal insufficiency, this metabolite can accumulate, raising the risk of drug toxicity (Afinogenova et al., 2022). Febuxostat commonly causes side effects such as liver dysfunction, diarrhea, headaches, nausea, vomiting, and rash (Terkeltaub, 2023; Schlesinger et al., 2023; Vargas-Santos and Neogi, 2017). This meta-analysis indicates that compared with the febuxostat groups, the allopurinol group (300 mg) has a lower incidence of cardiovascular events and renal abnormalities. Although benzbromarone is effective, it also has a relatively high risk of AEs. Due to the wide clinical application of benzbromarone, its clinical safety still needs to be further studied. In terms of drug use, as the dosage of ULTs increases, it is important to monitor for and promptly address any significant adverse reactions.

More precise strategies for drug treatment are expected to decrease the risk of adverse outcomes in gout patients. This study confirms that escalating the dose of medications like febuxostat, allopurinol, and benzbromarone in gout patients leads to a reduction in SUA levels and a decrease in the frequency of gout attacks. The dose-response relationship is analyzed in gout patients to explain the relationship between dose and SUA levels. Febuxostat and allopurinol may be the most effective conventional therapies for reducing SUA levels (Mackenzie et al., 2020; Zhang et al., 2021; Deng et al., 2024). Higher doses of allopurinol markedly increase the incidence of cardiovascular events and renal dysfunction, muscle and connective tissue events, and serious AEs. Moreover, this study confirms that increasing the dose of drugs such as febuxostat or allopurinol leads to a decrease in SUA levels and a reduction in the incidence of gout flares in patients. The relationship between dose and SUA levels is explained by the analysis of the dose-response relationship of gout patients. For reducing SUA levels, febuxostat and allopurinol may be the most effective conventional therapies (Becker et al., 2005b; Saag et al., 2022; Mu et al., 2019). An apparent increase in gastrointestinal, muscle, and connective tissue events linked to higher doses of allopurinol supports findings from previous studies on both allopurinol and febuxostat. In making more individualized treatment decisions, the benefits against the adverse effects and costs of these treatment options should be considered to promote the comprehensive development of ULT.

The current meta-analysis has some strengths. Firstly, we retrieve studies from Embase, PubMed, Cochrane Central Register of Controlled Trials, and Web of Science databases. 30 studies from different countries are included. This approach helps in identifying relevant information and reducing potential sampling errors. Secondly, according to the NOS, the included studies are of high quality, implying that the results of our meta-analysis are highly reliable. Thirdly, we perform dose-response analyses and generate linear dose-response curves, providing a quantitative estimate and visual graph of the association between high SUA levels and the risk of gout. Finally, this is the first NMA to compare the effects of different drug treatments in gout patients, and to rank all drugs based on their efficacy on SUA levels, gout flare, and AEs. This study provides valuable information for determining the optimal drug intervention for gout patients.

Nevertheless, this NMA has several limitations that should be acknowledged. Firstly, the small sample size may affect the accuracy and applicability of our results. Secondly, differences in treatment duration of patients in the included studies may lead to heterogeneity. Unfortunately, subgroup analyses could not be performed due to the limited number of studies. Lastly, the included studies are only published in English, introducing selection bias. Hence, more high-quality RCTs are required to confirm our findings.

## 5 Conclusion

The results of this NMA indicate that for the treatment of gout patients, taking 120 mg of febuxostat once daily has a noticeable advantage in achieving the target SUA level. As the dose increases, an increased risk of AEs is noted. In the future, more discussions on different dosage levels are needed to provide more precise treatment plans for clinical use. Our results offer new potential treatment strategies for gout. Subsequent studies can expand the range of inclusion and exclusion criteria or conduct targeted statistical analyses and meta-analyses of relevant articles on drugs for treating gout and related AEs.
