# Supplementary material for: Efficacy of different dosages of common uric acid-lowering medications in gout patients: a network meta-analysis of randomized control trials
Source: Front Pharmacol. 2025 Jun 25;16:1565530. doi: 10.3389/fphar.2025.1565530 (PMC12237641; doi:10.3389/fphar.2025.1565530)
Supplement: Supplementary file 1 [file DataSheet1.zip › Supplementary Table/S12 Heterogeneity analysis.docx]

| **Outcome** | **Comparison** | **Effect size（95%CrI）** | **I^2^(%)** |
| --- | --- | --- | --- |
| SUM | All300mg vs All100mg | -0.871(-1.789,0.05) | 94.8 |
|  | Feb40mg vs All 100mg | -1.421 (-2.303,-0.544) | 99.7 |
|  | Feb 40mg vs All 100mg | -1.169(-1.981,-0.357) | 99.7 |
|  | Feb 80mg vs All 100mg | -2.044(-2.983,-1.101) | 0 |
|  | Feb 40mg vs All 200 mg | -0.482(-1.602,0.640) | 0 |
|  | Placebo vs All 200mg | 3.2831(2.172,4.401) | 0 |
|  | Top_120mg vs All 200mg | -0.085(-1.130,0.965) | 0 |
|  | Feb 40mg vs All 300mg | -0.298(-0.857,0.254) | 93 |
|  | Feb 60mg vs All 300mg | -1.556(-2.784,-0.335) | 0 |
|  | Feb 80mg vs All 300mg | -1.175(-1.813,-0.532) | 88.9 |
|  | Placebo vs All 300mg | 3.469(2.548,4.381) | 0 |
|  | Feb 20mg vs Ben 25mg | 0.2419 (-0.644,1.127) | 30.6 |
|  | Feb 40mg vs Feb 20mg | -0.808(-1.821,0.213) | 94.7 |
|  | Placebo vs Feb 20mg | 2.959 (1.809,4.114) | 64.2 |
|  | Feb 60mg vs Feb 40mg | -1.258(-2.443,-0.076) | 0 |
|  | Feb 80mg vs Feb 40mg | -0.876(-1.471,-0.283) | 46.2 |
|  | Placebo vs Feb 40mg | 3.767 (2.939,4.590) | 0 |
|  | Placebo vs Feb 60mg | 5.022(3.830,6.222) | 0 |
|  | Placebo vs Feb 80mg | 4.643(3.699,5.586) | 0 |
|  | Top 120mg vs Placebo | -3.370(-4.655,-2.085) | 0 |
