# Supplementary material for: Efficacy of different dosages of common uric acid-lowering medications in gout patients: a network meta-analysis of randomized control trials
Source: Front Pharmacol. 2025 Jun 25;16:1565530. doi: 10.3389/fphar.2025.1565530 (PMC12237641; doi:10.3389/fphar.2025.1565530)

## S3: GRADE assessment for **SUA levels** NMA


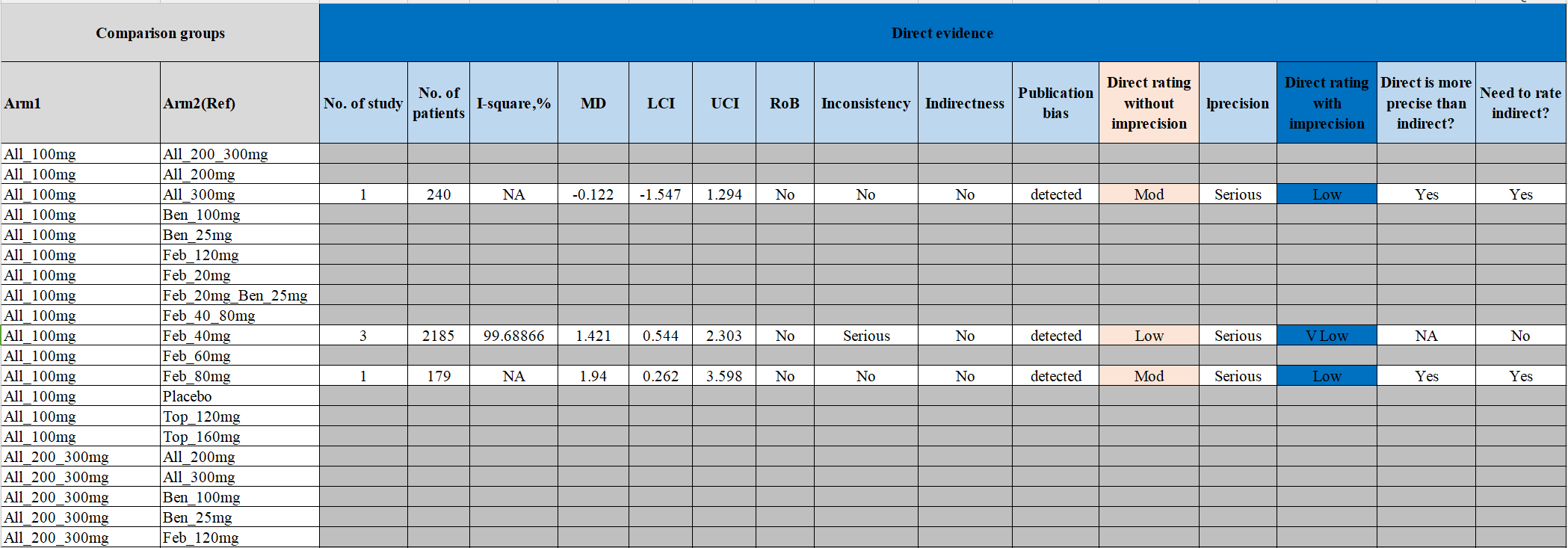


**S3 continued: GRADE assessment (indirect evidence) for SUA levels NMA**


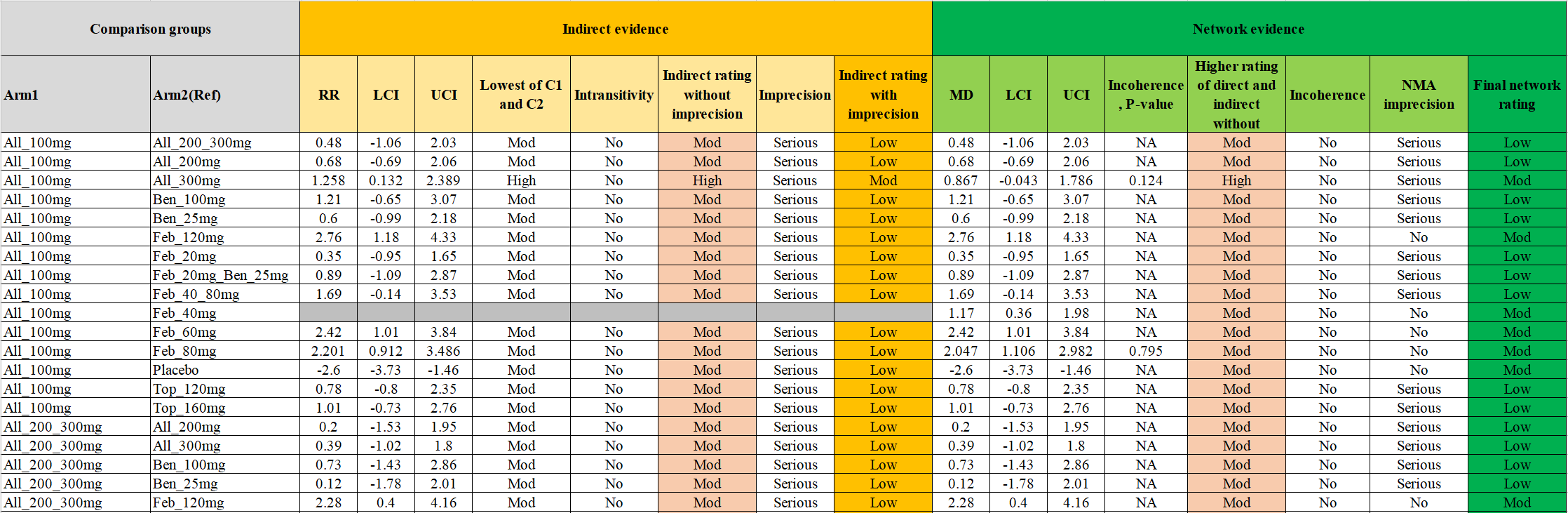


**S3 continued: GRADE assessment (direct evidence) for SUA levels NMA**


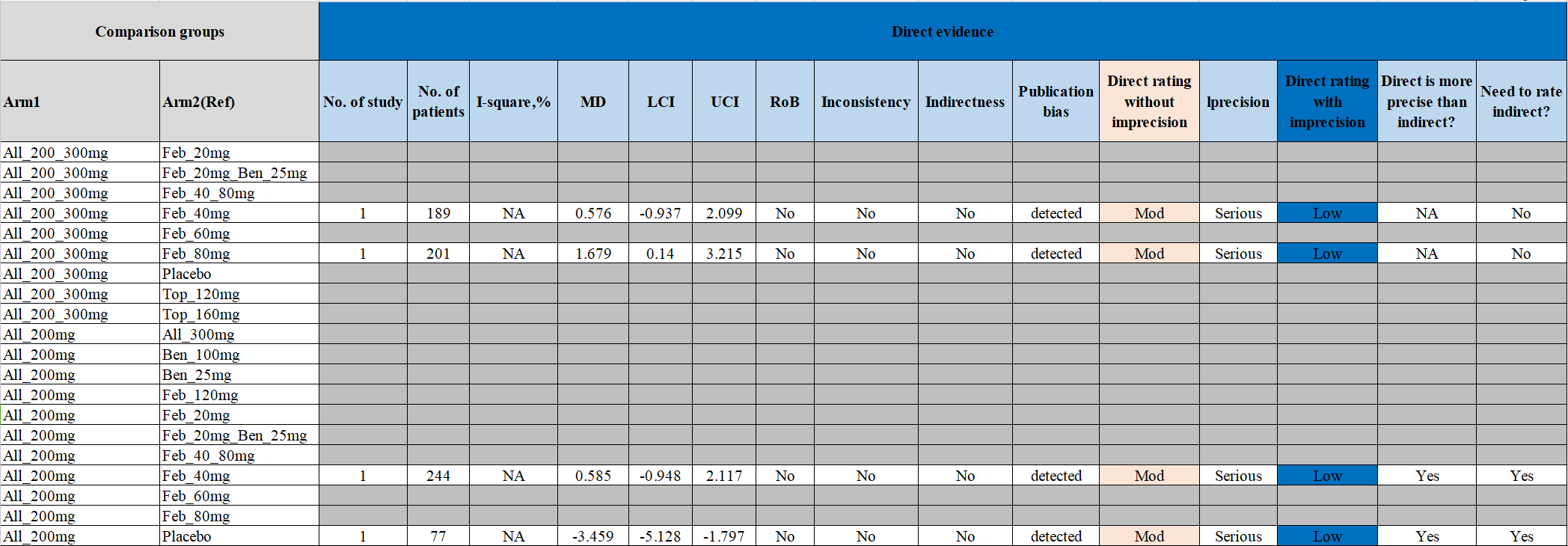


**S3 continued: GRADE assessment (indirect evidence) for SUA levels NMA**


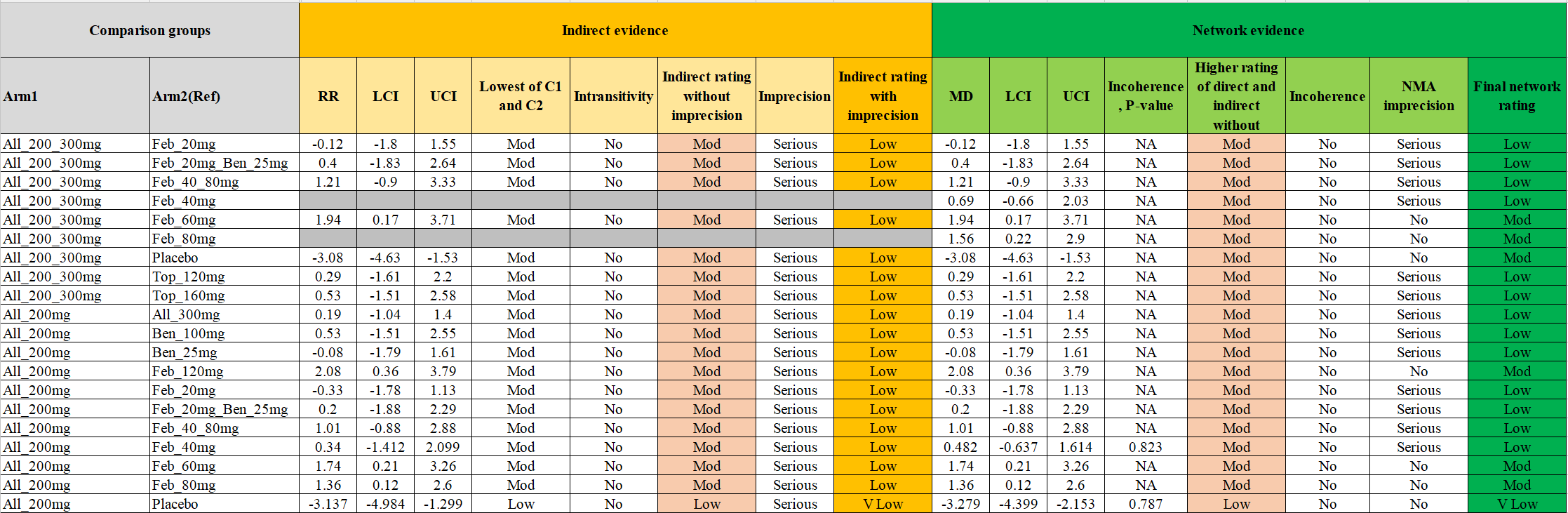


**S3 continued: GRADE assessment (direct evidence) for SUA levels NMA**


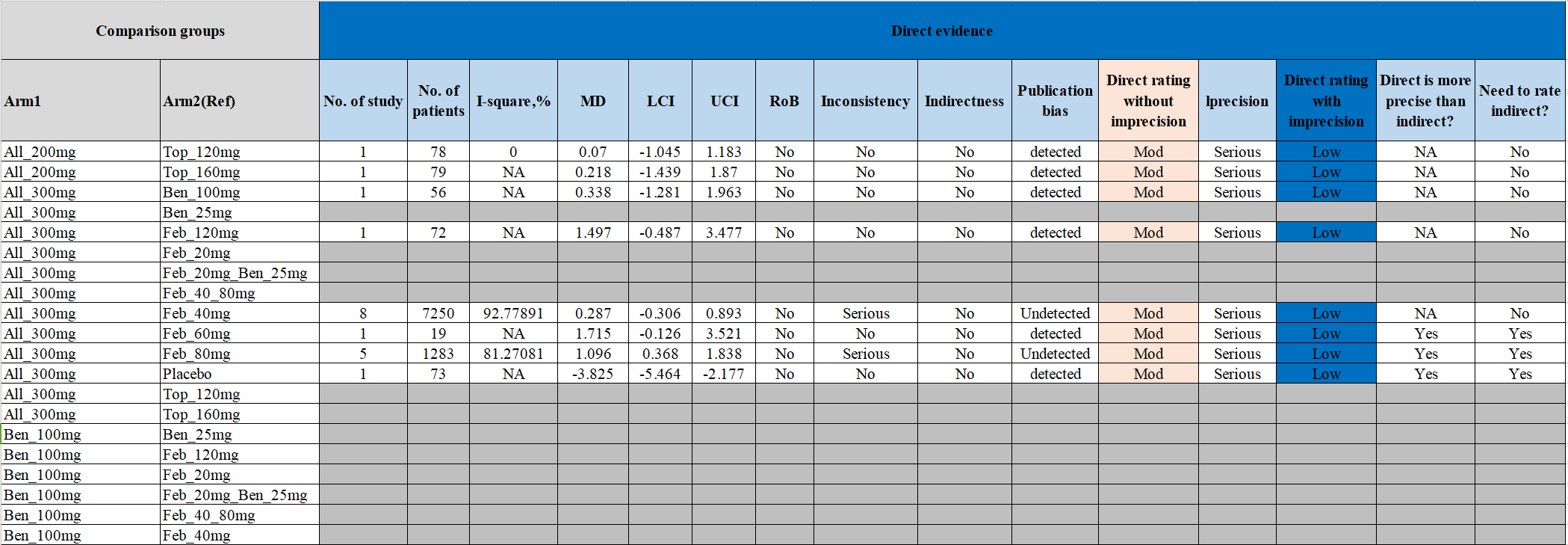


**S3 continued: GRADE assessment (indirect evidence) for SUA levels NMA**


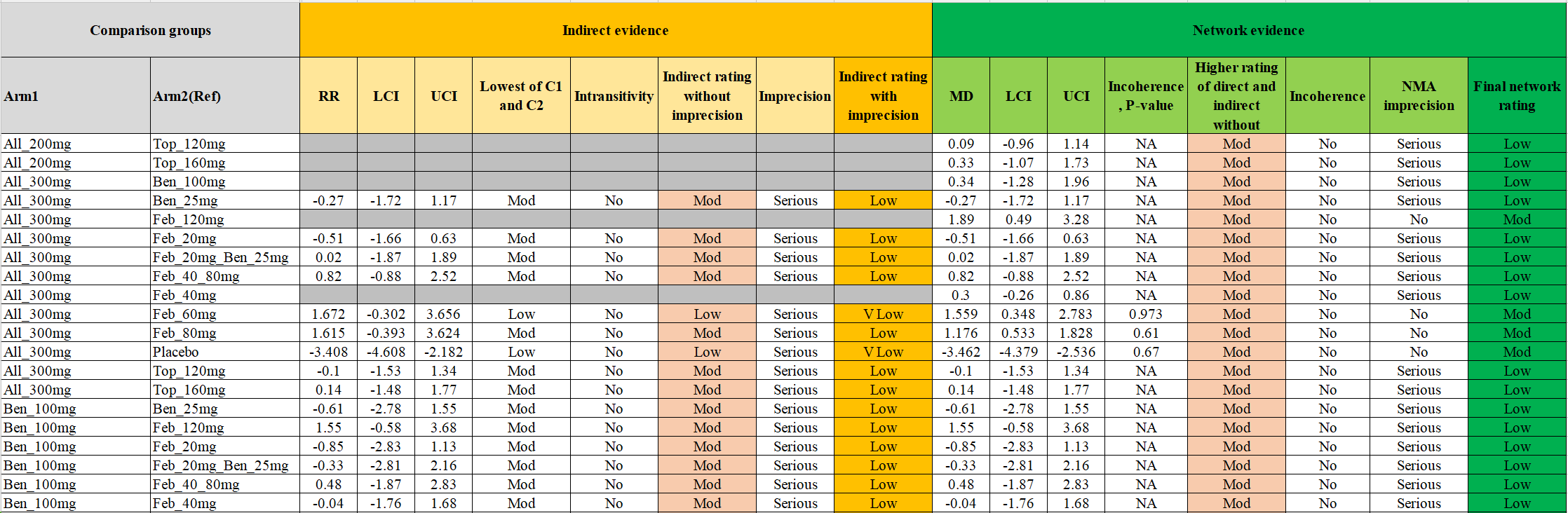


**S3 continued: GRADE assessment (direct evidence) for SUA levels NMA**


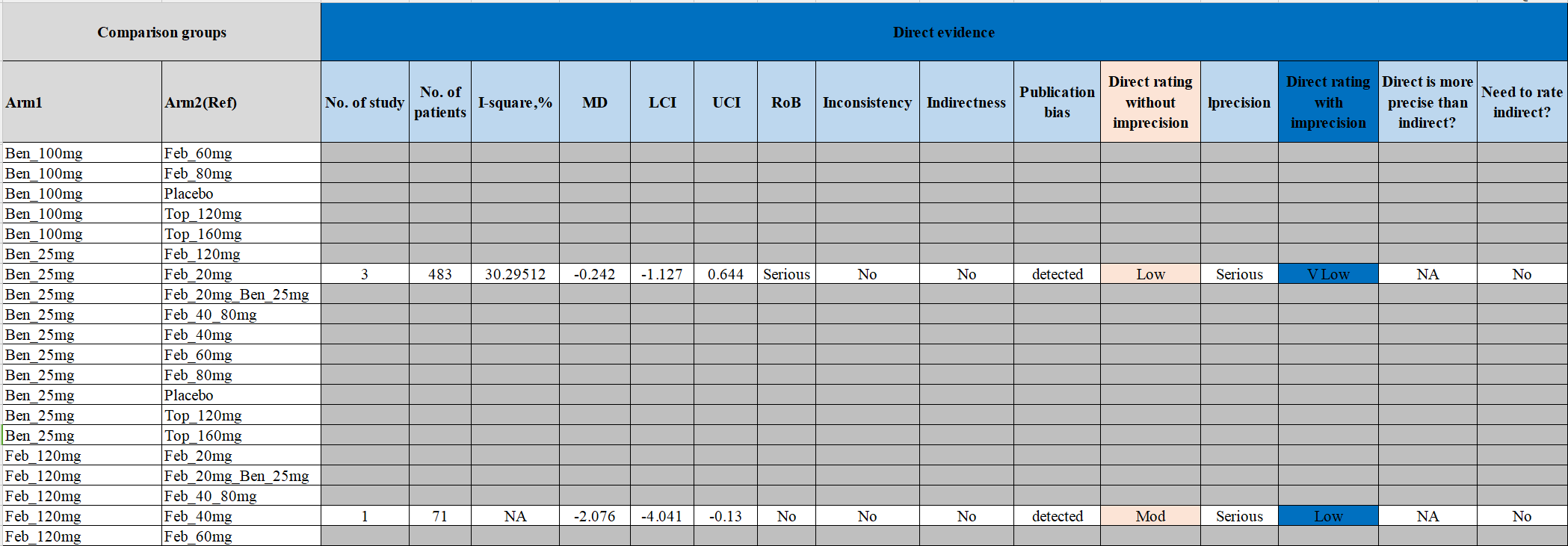


**S3 continued: GRADE assessment (indirect evidence) for SUA levels NMA**


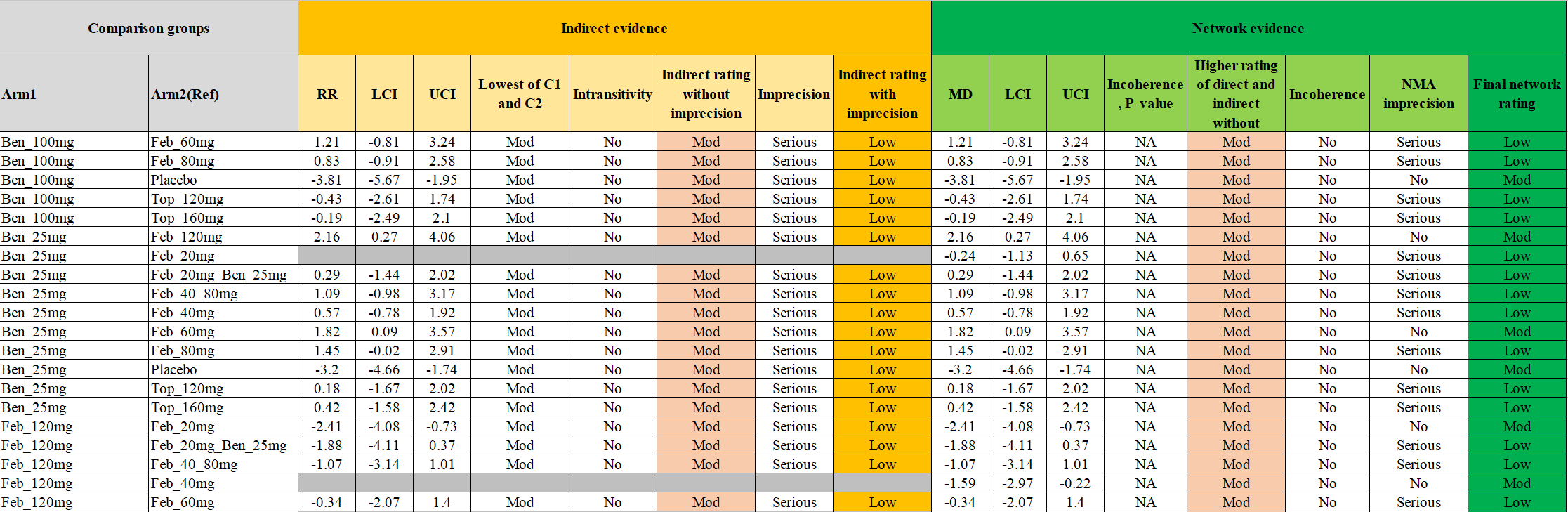


**S3 continued: GRADE assessment (direct evidence) for SUA levels NMA**


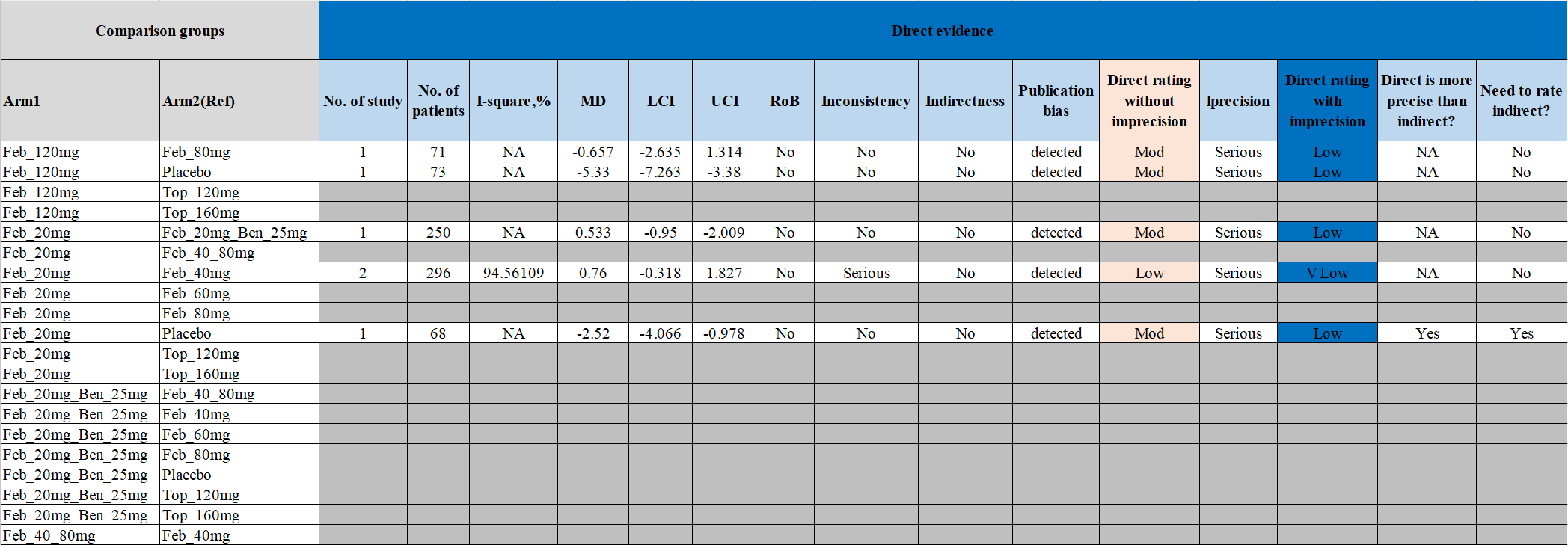


**S3 continued: GRADE assessment (indirect evidence) for SUA levels NMA**


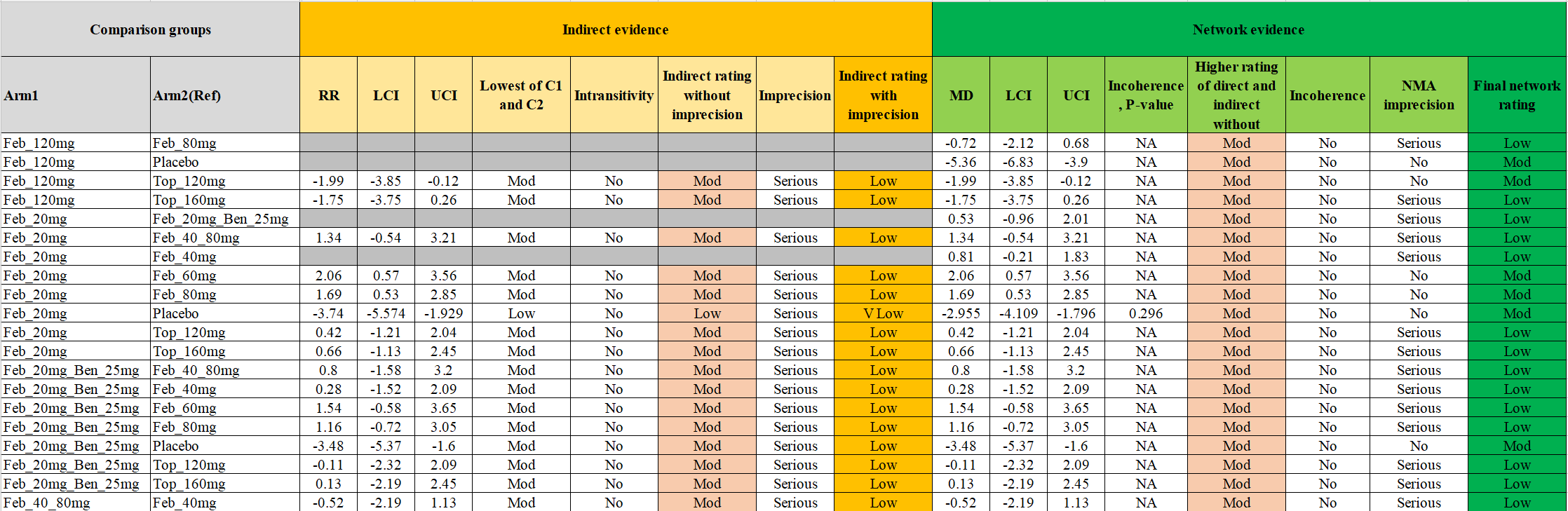


**S3 continued: GRADE assessment (direct evidence) for SUA levels NMA**


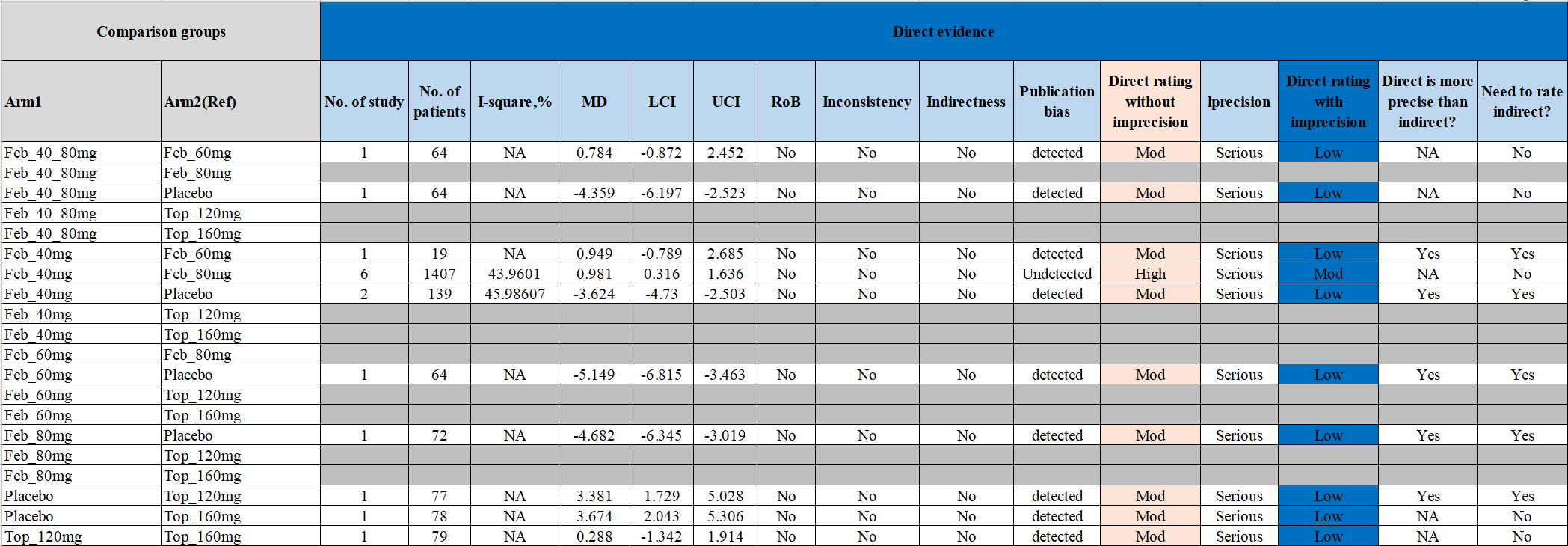


**S3 continued: GRADE assessment (direct evidence) for SUA levels NMA**


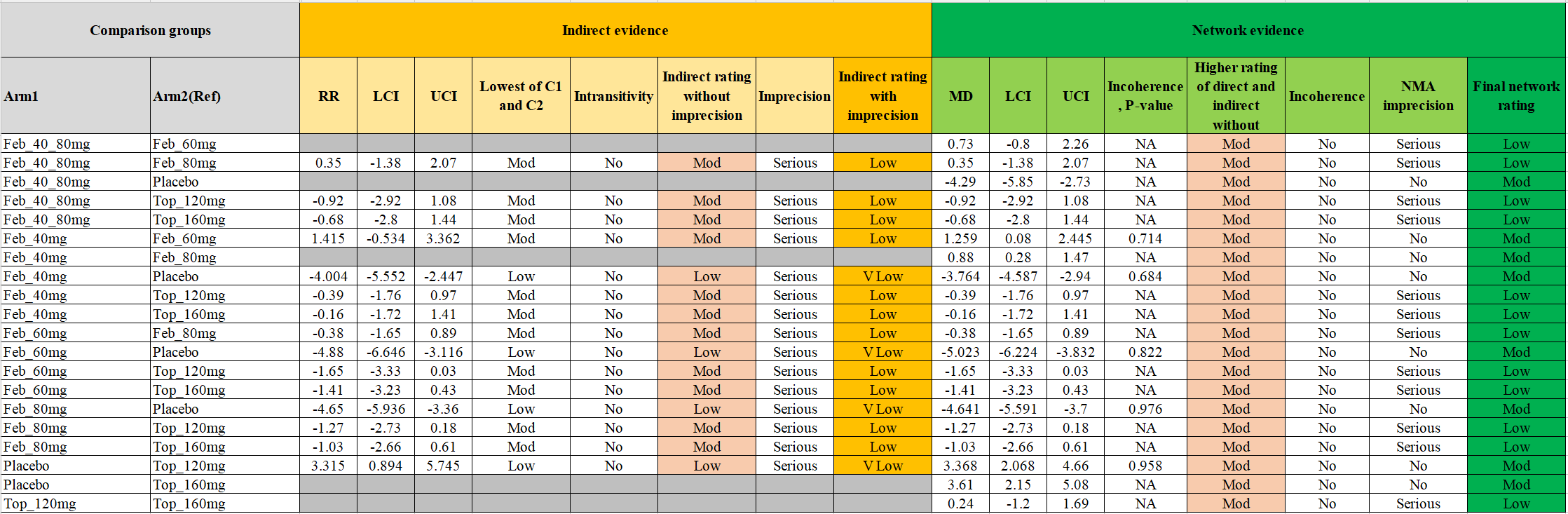

Supplement: Supplementary file 1 [file DataSheet1.zip › Supplementary Table/S3 GRADE.docx]
