# Supplementary figures and images for: Efficacy of different dosages of common uric acid-lowering medications in gout patients: a network meta-analysis of randomized control trials
Source: Front Pharmacol. 2025 Jun 25;16:1565530. doi: 10.3389/fphar.2025.1565530 (PMC12237641; doi:10.3389/fphar.2025.1565530)

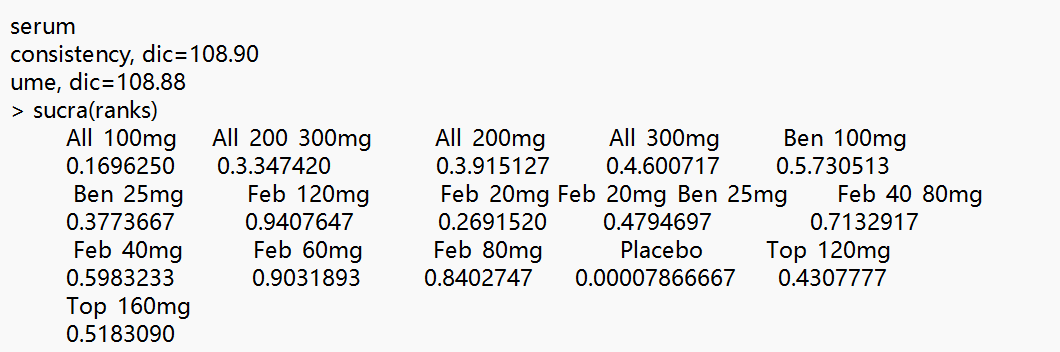

Supplement: Supplementary file 1 [file DataSheet1.zip › Supplementary Table/S1.png]

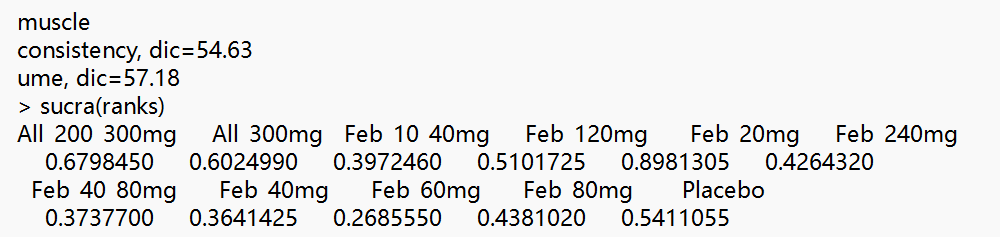

Supplement: Supplementary file 1 [file DataSheet1.zip › Supplementary Table/S10.png]

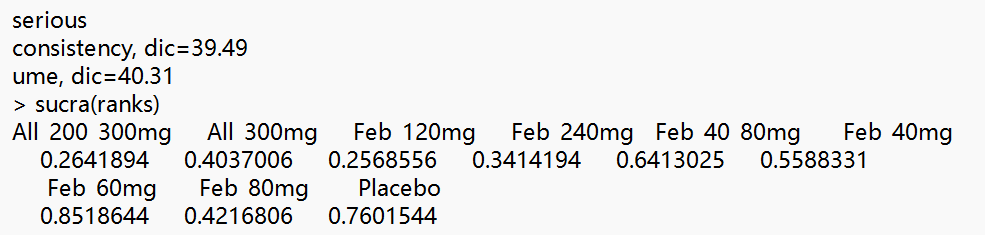

Supplement: Supplementary file 1 [file DataSheet1.zip › Supplementary Table/S11.png]

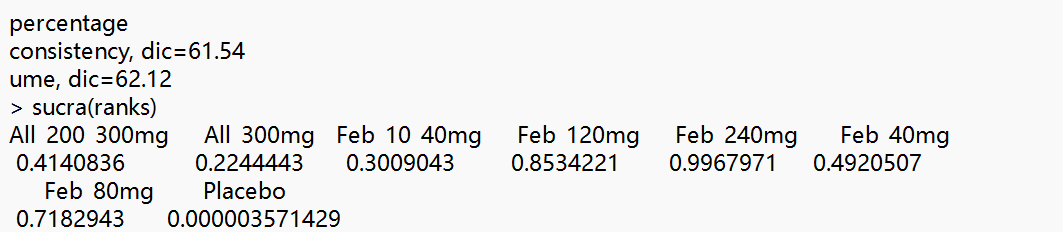

Supplement: Supplementary file 1 [file DataSheet1.zip › Supplementary Table/S2.png]

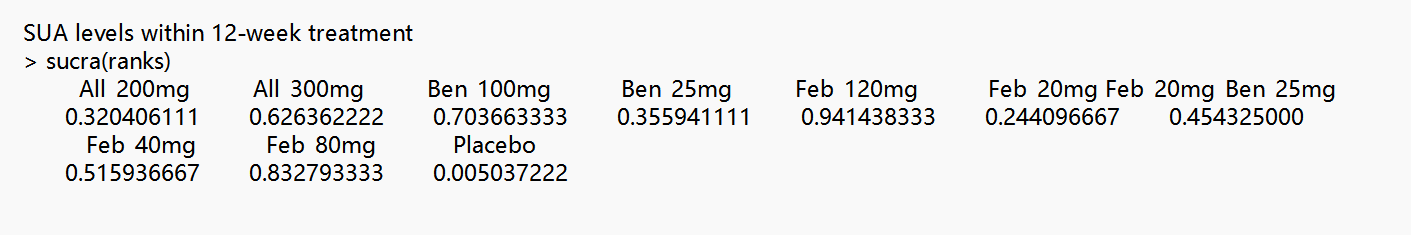

Supplement: Supplementary file 1 [file DataSheet1.zip › Supplementary Table/S4.png]

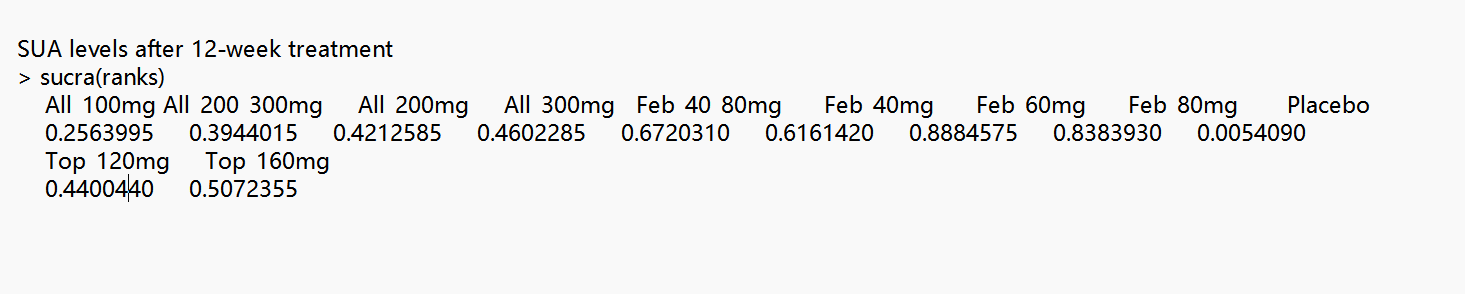

Supplement: Supplementary file 1 [file DataSheet1.zip › Supplementary Table/S5.png]

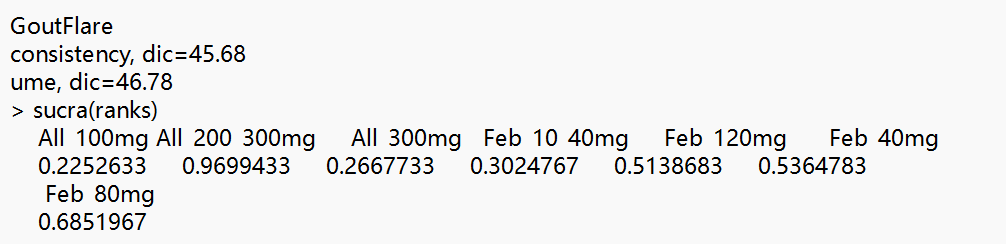

Supplement: Supplementary file 1 [file DataSheet1.zip › Supplementary Table/S6.png]

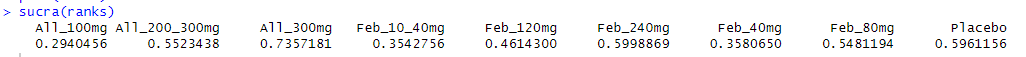

Supplement: Supplementary file 1 [file DataSheet1.zip › Supplementary Table/S7.png]

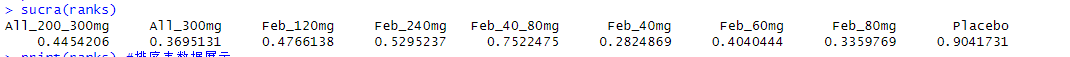

Supplement: Supplementary file 1 [file DataSheet1.zip › Supplementary Table/S8.png]

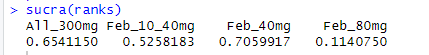

Supplement: Supplementary file 1 [file DataSheet1.zip › Supplementary Table/S9.png]
